# Genome-Wide Single Nucleotide Polymorphism Analysis Elucidates the Evolution of *Prunus takesimensis* in Ulleung Island: The Genetic Consequences of Anagenetic Speciation

**DOI:** 10.3389/fpls.2021.706195

**Published:** 2021-09-02

**Authors:** Myong-Suk Cho, Koji Takayama, JiYoung Yang, Masayuki Maki, Seung-Chul Kim

**Affiliations:** ^1^Department of Biological Sciences, Sungkyunkwan University, Suwon, South Korea; ^2^Department of Botany, Graduate School of Science, Kyoto University, Kyoto, Japan; ^3^Research Institute for Dok-do and Ulleung-do Island, Kyungpook National University, Daegu, South Korea; ^4^Botanical Gardens, Tohoku University, Sendai, Japan

**Keywords:** wild flowering cherry, MIG-seq analysis, chlorotype network, *Prunus takesimensis*, *Prunus sargentii*, Ulleung Island

## Abstract

Of the two major speciation modes of endemic plants on oceanic islands, cladogenesis and anagenesis, the latter has been recently emphasized as an effective mechanism for increasing plant diversity in isolated, ecologically homogeneous insular settings. As the only flowering cherry occurring on Ulleung Island in the East Sea (concurrently known as Sea of Japan), *Prunus takesimensis* Nakai has been presumed to be derived through anagenetic speciation on the island. Based on morphological similarities, *P. sargentii* Rehder distributed in adjacent continental areas and islands has been suggested as a purported continental progenitor. However, the overall genetic complexity and resultant non-monophyly of closely related flowering cherries have hindered the determination of their phylogenetic relationships as well as the establishment of concrete continental progenitors and insular derivative relationships. Based on extensive sampling of wild flowering cherries, including *P. takesimensis* and *P. sargentii* from Ulleung Island and its adjacent areas, the current study revealed the origin and evolution of *P. takesimensis* using multiple molecular markers. The results of phylogenetic reconstruction and population genetic structure analyses based on single nucleotide polymorphisms detected by multiplexed inter-simple sequence repeat genotyping by sequencing (MIG-seq) and complementary cpDNA haplotypes provided evidence for (1) the monophyly of *P. takesimensis*; (2) clear genetic differentiation between *P. takesimensis* (insular derivative) and *P. sargentii* (continental progenitor); (3) uncertain geographic origin of *P. takesimensis*, but highly likely via single colonization from the source population of *P. sargentii* in the Korean Peninsula; (4) no significant reduction in genetic diversity in anagenetically derived insular species, i.e., *P. takesimensis*, compared to its continental progenitor *P. sargentii*; (5) no strong population genetic structuring or geographical patterns in the insular derivative species; and (6) MIG-seq method as an effective tool to elucidate the complex evolutionary history of plant groups.

## Introduction

**FIGURE 1:**
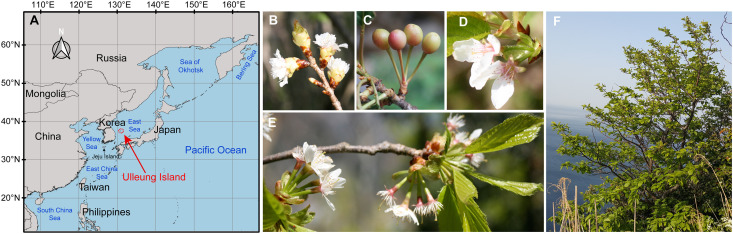
A map showing the location of Ulleung Island **(A)**, and photographs of the wild flowering cherry, *Prunus takesimensis*, endemic to Ulleung Island, South Korea **(B–F)**.

The genus *Prunus* comprises approximately 200 species of shrubs and trees (Rehder, 1940; Kalkman, 2004), including many economically important fruit trees (e.g., almonds, apricots, cherries, peaches, and plums) as well as ornamental, medicinal, and timber species (Ingram, 1948; Elias, 1980; Zomlefer, 1994; Potter, 2011). Flowering cherries are one of the most popular ornamentals and cultivated trees worldwide, and are classified under the subgenus *Cerasus* of the genus *Prunus*, which is native to temperate Asia, Europe, and North America (Li and Bartholomew, 2003). Many forms of ornamental flowering cherries with diverse origins and traits have been cultivated from a wide range of wild flowering cherry species growing in the forests of eastern Asia. *Prunus takesimensis* Nakai is a wild flowering cherry that is endemic to Ulleung Island, South Korea (see Figure 1A for its location). Given its sole representation on the island, *P. takesimensis* has purportedly originated via anagenetic speciation from a continental progenitor species (Sun and Stuessy, 1998; Stuessy et al., 2006). Ulleung Island is located in the East Sea/Sea of Japan between 130°47′E–131°52′E longitude and 37°33′N–37°14′N latitude, with the shortest distance of 137 km from the east coast of the Korean Peninsula. The island is of volcanic origin and approximately 1.8 million years (Myr) old; it has never been connected to the adjacent continental land mass (Kim, 1985). Despite its relatively small size (total area of approximately 73 km^2^ with the highest peak being 984 m above sea level), Ulleung Island is rich in flora with approximately 500 native vascular plant species, of which approximately 37 are endemic (Lee and Yang, 1981). Most of these endemic species are single representatives of diverse vascular plant families that might have derived anagenetically from continental progenitors in adjacent source areas (Sun and Stuessy, 1998).

The most commonly described evolutionary process in island biogeography is speciation associated with cladogenesis via adaptive radiation (Carlquist, 1974; Stuessy et al., 2006; Stuessy, 2007). Cladogenetic speciation is generally known to involve an initial immigrant population splitting into morphologically and ecologically distinct evolutionary lineages through adaptation to divergent habitats on the island, resulting in two or more new species being recognized taxonomically. There are numerous examples of this speciation mode, including the *Lobelia* complex (Campanulaceae) (Givnish et al., 2009) and the silversword alliance (Carlquist et al., 2003) in Hawaii; *Scalesia* (Asteraceae) in the Gálapagos Islands (Eliasson, 1974; Schilling et al., 1994; Fernández-Mazuecos et al., 2020); *Echium* (Boraginaceae) (Böhle et al., 1996; Garcıa-Maroto et al., 2009), *Aeonium* (Crassulaceae) (Jorgensen and Olesen, 2001; Mort et al., 2002), and the woody *Sonchus* alliance (Asteraceae) (Kim et al., 1996a, b, 2008; Santiago and Kim, 2009) in the Canary Islands; and *Dendroseris* and *Robinsonia* (Asteraceae) in the Juan Fernández Islands (Crawford et al., 1998; Cho et al., 2020). In addition to the adaptive radiation in heterogeneous habitats, examples of cladogenetic speciation triggered by non-adaptive radiation have been also presented, such as *Nigella arvensis* complex (Ranunculaceae) in the Aegean Archipelago (Comes et al., 2008; Jaros et al., 2018) and *Helianthemum* (Cistaceae) in the Canary Islands (Albaladejo et al., 2021). In non-adaptive radiation, speciation not accompanied by relevant adaptation into various niches precedes significant ecological differentiation through geographical isolation, resulting in allopatric speciation (Rundell and Price, 2009).

A different mode of evolution, anagenetic speciation, which describes lineages changing over time without splitting events on islands, has been reported, explaining ca. 25% of insular endemic angiosperm species diversity (Stuessy et al., 2006; Takayama et al., 2015). In anagenetic speciation, a founder population arriving on an oceanic island proliferates in a favorable uniform environment and spreads over the island, gradually accumulates genetic variation through mutation and recombination in isolated environments, and eventually diverges from continental source populations in genetic composition and morphological characteristics (Stuessy et al., 2006, 2014; Stuessy, 2007; Takayama et al., 2015). Unlike adaptive radiation, the investigation of anagenetic speciation has been limited to a few geographical regions, primarily to the Juan Fernández Islands in the Pacific Ocean (López-Sepúlveda et al., 2013, 2015; Takayama et al., 2015) and Ulleung Island in East Asia (Pfosser et al., 2005; Takayama et al., 2012, 2013). The expected genetic outcomes of speciation via cladogenesis or anagenesis are different. In cladogenesis, morphological or ecological divergence among species is often notable owing to dispersal into different environments and strong selection, but the overall genetic differentiation among populations is generally low within a complex of closely related species. In contrast, lack of geographic partitioning of genetic variations maintains high genetic diversity levels in anagenetic speciation, and no or weak geographical genetic structure is found among the island populations of anagenetically derived species (Stuessy et al., 2006, 2014; Takayama et al., 2015).

Ulleung Island is of particular interest to evolutionary biologists and phytogeographers. It is known for the exceptionally high level of anagenetic speciation; 88% of the total endemic plants are anagenetically derived. In comparison, rates of anagenetic speciation among endemic plants on other oceanic islands, such as Hawaii, Bonin Islands, and St. Helena, are 7, 53, and 53%, respectively (Stuessy et al., 2006). Ulleung Island is young (approximately 1.8 Myr old) (Kim, 1985), of low elevation (<1000 m), and relatively ecologically uniform (Yim et al., 1981), and these factors are known to be correlated with a high frequency of anagenetic speciation. Only a few endemic species on Ulleung Island have been investigated to determine continental progenitor species and better understand the genetic consequences of anagenetic speciation. Recently, three endemic plants, *Rubus takesimensis* Nakai (Yang et al., 2019), *Campanula takesimana* Nakai (Cheong et al., 2020), and *Phedimus takesimensis* (Nakai) ’t Hart (Seo et al., 2020) have been investigated based on maternally inherited plastid DNA sequences, whereas *Dystaenia takesimana* (Nakai) Kitag. (Pfosser et al., 2005), *Acer okamotoanum* Nakai (Pfosser et al., 2002; Takayama et al., 2012), and *Acer takesimense* Nakai (Pfosser et al., 2002; Takayama et al., 2013) have been previously investigated based on nuclear microsatellite and amplified fragment length polymorphism markers. Their respective genetic patterns in geographic source areas and genetic variations appear to be complex. It has been demonstrated that *D. takesimana*, *A. takesimense*, and *A. okamotoanum* show higher or slightly lower levels of genetic variation than their continental progenitor species (Pfosser et al., 2002, 2005; Takayama et al., 2012, 2013). However, the populations of *R. takesimensis* on Ulleung Island show significantly lower genetic diversity than its continental progenitor, *R. crataegifolius*, without geographical population structuring on the island (Yang et al., 2019). Similarly, *C. takesimana* is substantially less genetically diverse than its continental progenitor, *C. punctata*, sampled from the Korean Peninsula, but it shows significant population genetic structuring (Cheong et al., 2020). *Phedimus takesimensis* also shows apparent genetic structuring within Ulleung Island, presumably owing to its limited seed dispersal mechanism, without apparent reduction in genetic diversity (Seo et al., 2020). To further assess the evolutionary importance and emerging patterns of anagenetic speciation on oceanic islands, it is necessary to explore more diverse anagenetically derived endemic species on Ulleung Island using variable molecular markers.

Of the endemic species purportedly derived via anagenesis on Ulleung Island, *Prunus takesimensis* Nakai is an exceptionally challenging taxon to elucidate its origin and evolution. It suffers from the difficulties in species delimitation and unresolved phylogenetic relationships of flowering cherries due to morphological continuity, lack of diagnostic morphological features, limited informative genetic polymorphisms from appropriate molecular markers, and frequent hybridization and introgression among congeneric species (Bortiri et al., 2001, 2002; Potter, 2011; Chin et al., 2014; Cho et al., 2014; Cho and Kim, 2019). *P. takesimensis* is a deciduous tree species growing up to 20 m high with coetaneous flowering in April and fruiting in late May through June. It is commonly found in wild forests and also cultivated as a popular ornamental tree in residential landscapes, gardens, parks, and streets in Ulleung Island. *P. takesimensis* was described by Nakai (1918) based on several diagnostic features, such as umbellate inflorescence with 2–5 flowers, absence of hairs in leaves, pedicels and petioles, and coetaneous phenology. However, most of these features are shared with the congeneric species, *Prunus sargentii* Rehder, which is currently considered the most likely candidate progenitor of *P. takesimensis*. *Prunus sargentii* is usually found in the high mountains of the eastern Korean Peninsula along the Baekdudaegan Mountain Range, Jeju Island, Hokkaido and Honshu in northern Japan, and the Russian Far East (Ohwi, 1984; Chang et al., 2004; Kim, 2009). Using multivariate morphometric analyses of morphological characteristics, Chang et al. (2004) identified *P. takesimensis* as a cohesive group distinct from *P*. *sargentii* based on smaller flower size (diameter: 26–32 mm vs. 34–48 mm, respectively) and higher flower numbers per umbellate inflorescence (3–5 flowers vs. 2–3 flowers, respectively) (Figures 1B–F). Additionally, *P. takesimensis* has entire and erect (or spreading) calyx lobes and lacks hair on the bud scale, inflorescence, leaf, petiole, and pedicel; however, rare individuals with hair on the pedicel can be found (Cho, M.-S., personal observation; Figure 1D).

Despite its morphological distinction from congeneric flowering cherries, *P. takesimensis* has never been resolved as a monophyletic lineage in any prior molecular phylogenetic analyses. In addition, no comprehensive study has been conducted to determine its progenitor-derivative relationship or population genetic diversity and structure. For example, with the inclusion of limited samples of *P. takesimensis*, phylogenetic analyses of *Prunus*/*Cerasus* have been conducted to determine primary interspecific relationships (Bortiri et al., 2001; Jung and Oh, 2005; Cho and Kim, 2019). Simple sequence repeat (SSR) genotyping of the collections of ornamental *Prunus* germplasm at the United States National Arboretum (USNA) suggested genetic closeness between *P. takesimensis* and *P*. *sargentii*, although both species were not retrieved as monophyletic (Ma et al., 2009). Cho et al. (2014) and Cho and Kim (2019), based on nuclear ribosomal DNA (nrDNA) internal (ITS) and external transcribed spacer (ETS) and chloroplast non-coding regions, attempted to resolve interspecific relationships within *Prunus* in South Korea. However, the resulting phylogenetic trees were poorly resolved since most nodes in the trees were not well-supported. Several phenomena, such as recent speciation and cross-compatibility among species, historical and contemporary gene flow, and incomplete lineage sorting of ancestral polymorphisms, further complicate our understanding of the evolution of flowering cherries (Ohta et al., 2007).

Given the abovementioned methodological challenges for the study of the evolutionary processes of flowering cherries in a phylogenetic framework, we performed a genome-wide single nucleotide polymorphism (SNP) analysis using multiplexed inter-SSR (ISSR) genotyping by sequencing (MIG-seq) in addition to Sanger-derived sequences for nrDNA (ITS and ETS) and seven concatenated cpDNA non-coding regions. MIG-seq is a polymerase chain reaction (PCR)-based next-generation sequencing (NGS) method, which has been recently developed as an effective method for the discovery of genome-wide SNPs from low-quantity or low-quality DNA (Suyama and Matsuki, 2015). It has been used successfully and effectively to clarify persistent taxonomic difficulties or issues for several plant groups with complex evolutionary history based on genome-wide SNPs (Binh et al., 2018; Gutiérrez-Ortega et al., 2018; Yoichi et al., 2018; Park et al., 2019, 2020; Takata et al., 2019; Strijk et al., 2020; Nakamura et al., 2021; Onosato et al., 2021). In addition to determining the interspecific relationships based on the genome-wide SNPs, we compared the genetic diversity and population genetic structure between the insular derivative, *P*. *takesimensis*, and the purported continental ancestor, *P. sargentii*, using chloroplast DNA (cpDNA) as a maternally inherited marker. It has been hypothesized that *P. takesimensis* on Ulleung Island has been originated from its continental progenitor species, *P. sargentii* according to the common morphological characteristics (Chang et al., 2004). However, this hypothesis has never been rigorously tested based on a broad and suitable sampling strategy using highly variable molecular markers. Therefore, in the present study, we extensively sampled *P*. *takesimensis* from Ulleung Island and all closely related flowering cherry species from adjacent regions [14 populations (189 accessions) of *P*. *takesimensis* and 161 of other species in the subgenus *Cerasus*]. In particular, the purported continental progenitor species, *P. sargentii*, was sampled from likely source areas, such as the Korean Peninsula, including Jeju Island, Japan, and the Russian Far East, to determine the geographical source area. The primary objectives of this study were to (1) test the monophyly of *P. takesimensis* on Ulleung Island and resolve its sister group relationship for identifying its continental progenitor species and source populations in the phylogenetic framework employing genome-wide MIG-seq analysis as well as Sanger sequencing for nrDNA regions ITS and ETS, and seven concatenated cpDNA regions. Secondly, we performed population genetic analyses to (2) evaluate the patterns of genetic variation within the insular populations of *P. takesimensis* and compare them with the results in its continental progenitor species using MIG-seq and cpDNA data. These objectives allow us to better understand the origin of Ulleung Island endemic plants as well as the genetic consequences of anagenetic speciation in the East Sea.

## Materials and Methods

### Plant Material and DNA Isolation

This study followed the most widely accepted Rehder’s classification(1940), where the genus *Prunus* is broadly interpreted and divided into five subgenera. Leaves collected mostly from natural populations were dried with silica gel and used as DNA sources. DNA was extracted using the DNeasy Plant Mini Kit (Qiagen, Carlsbad, CA, United States). We included extensive samples in analyses, a total of 350 accessions belonging to 15 *Prunus* species in the subgenus *Cerasus* (Supplementary Table 1). First, 189 accessions from 14 populations of *P. takesimensis* were sampled from Ulleung Island in addition to 161 accessions of other flowering cherries sampled from Jeju Island, the Korean Peninsula, the Russian Far East, and Japan. Twelve taxa belonging to the subgenus *Cerasus*, section *Pseudocerasus*, were sampled; *P. spachiana* f. *ascendens* (four accessions: three from South Korea and one from Japan), *P. sargentii* Rehder (65 accessions: 36 from South Korea, seven from the Russian Far East, and 22 from Japan), *P. sargentii* var. *verecunda* (Koidz.) Chin S. Chang (four accessions from South Korea), *P. serrulata* var. *spontanea* (Max) Wilson (21 accessions: 16 from South Korea and five from Japan), *P. serrulata* var. *quelpaertensis* (Nakai) Uyeki (10 accessions from Jeju Island, South Korea), *P. serrulata* var. *pubescens* (Makino) Nakai (17 accessions: 16 from South Korea and one from Japan), *P. yedoensis* var. *angustipetala* Kim and Kim (one accession from Jeju Island), *P. hallasanensis* Kim & Kim (two accessions from Jeju Island), *P. longistylus* Kim and Kim (one accession from Jeju Island), *P. speciosa* (Koidz.) Ingram (28 accessions: three cultivated accessions from Jeju Island, South Korea, and 25 wild accessions from Japan), *P*. *incisa* Thunb. (one accession from Japan), and *P*. *apetala* (Siebold and Zucc.) Franch and Sav. (three accessions from Japan). Two taxa belonging to the subgenus *Cerasus*, section *Phyllomahaleb* and section *Eucerasus* were also included: *P. maximowiczii* Ruprecht (three accessions from South Korea) and *P*. *avium* (L.) L. (one accession from Japan).

Of a total of 350 accessions, 123 accessions representing 13 *Cerasus* species were used for phylogenetic analyses based on nrDNA ITS and ETS as well as seven concatenated cpDNA non-coding regions. In total, 262 accessions from 13 species, including *P. takesimensis* (162 accessions), *P. sargentii* (46 accessions), and other species (54 accessions) were used to determine the phylogenetic position and genetic structure of *P. takesimensis* by MIG-seq SNP analysis. Ninety nine accessions of *P. takesimensis* and *P. sargentii* sampled at the population level were used for the analysis of the cpDNA haplotype network (Supplementary Table 1). Voucher specimens were deposited at the Ha Eun Herbarium, Sungkyunkwan University (SKK), South Korea.

### nrDNA and cpDNA Sequences

Nuclear ITS and ETS DNA regions and seven highly variable non-coding regions of chloroplast DNA (*pet*A-*psb*J, *pet*D-*rpo*A, *ndh*F-*rpl*32, *trn*Q-*rps*16, *trn*V-*ndh*C, *rpl*16 intron, and *trn*L-*rpl*32) (Shaw et al., 2007) were amplified for phylogenetic analyses of 123 accessions of 14 *Cerasus* species (Supplementary Table 1). These datasets were part of our earlier studies (Cho et al., 2014; Cho and Kim, 2019), which were limited to the ranges of the species belonging to *P. serrulata*/*P. sargentii* complex and other closely related species. Four accessions of *P. spachiana* f. *ascendens* collected from Japan and South Korea were included as outgroup. To gain additional insights into the phylogenetic relationships between continental progenitors and insular derivative species pairs, we produced a population-level cpDNA data matrix (including five to eight accessions per population) for *P. takesimensis* and *P. sargentii* using five non-coding regions (*pet*A-*psb*J, *pet*D-*rpo*A, *trn*Q-*rps*16, *rpl*16 intron, and *trn*L-*rpl*32; Shaw et al., 2007). The dataset included 13 populations of *P. takesimensis* from Ulleung Island and five populations of *P. sargentii* collected from its adjacent areas, comprising a total of 99 accessions (see Table 1 for the sampling localities and numbers of each population). All primer pairs used for amplification were as specified in our previous studies (Cho et al., 2014; Cho and Kim, 2019). The thermal cycler program was run as follows: one cycle of 95°C for 2 min (initial denaturation), 35 cycles of 20 s at 95°C (denaturation), 40 s at 52°C (annealing), 1 min at 72°C (extension), and finally 5 min at 72°C (final extension). All PCR products were purified using the Inclone Gel & PCR Purification Kit (InClone Biotech Co., Seoul, South Korea). Direct sequencing of the purified PCR products was carried out using the BigDye Terminator v3.1 Cycle Sequencing Kit (Applied Biosystems, Foster City, CA, United States) at the Geno Tech Corp. (Daejeon, South Korea). Contig assembly was made using Mafft ver. 7.017 (Katoh et al., 2002) with default parameters (Auto Algorithm, 200PAM/k = 2 Scoring matrix, 1.53 Gap open penalty and 0.123 Offset value), and editing was performed manually by using Geneious ver. 8.1.7 (Kearse et al., 2012).

**TABLE 1:** List of numbers and sampling locations of *P. takesimensis* and *P. sargentii* used for population-level analyses of cpDNA haplotype and MIG-seq SNPs performed in this study.

Population code	Locality	GPS	Altitude (m)	N/I cpDNA network	N/I SNP (MIG-seq) analysis
*Prunus takesimensis* Nakai					
CBU	Cheonbu, Ulleung Island, South Korea	37°32′17.4′′N 130°52′53.0′′E	131 m	5 individuals	10 individuals
CHU	Chusan, Ulleung Island	37°31′31.7′′N 130°51′26.6′′E	283 m	5	14
DDJ	Dokdo Jeonmangdae, Ulleung Island	37°28′47.8′′N 130°54′15.1′′E	247 m	5	12
HGM	Hyangmok Jeonmangdae, Ulleung Island	37°30′55.0′′N 130°47′53.0′′E	138 m	5	14
HPR	Hyunpo-ri, Ulleung Island	37°31′19.8′′N 130°49′10.4′′E	86 m	5	12
JRG	Joongryong, Ulleung Island	37°27′50.97′′N 130°52′05.10′′E	250 m	5	14
MAL	Maljandeung, Ulleung Island	37°30′38.8′′N 130°53′07.3′′E	737 m	5	10
NAM	Namseo-ri, Ulleung Island	37°29′19.0′′N 130°50′02.3′′E	248 m	5	9
NAR	Nari, Ulleung Island	37°30′33.9′′N 130°51′41.8′′E	443 m	6	11
NRH*	Nari, Ulleung Island			0	10
NSJ	Naesujeon, Ulleung Island	37°30′47.0′′N 130°54′25.3′′E	341 m	5	5
SAD	Sa-dong, Ulleung Island	37°28′44.5′′N 130°52′18.3′′E	347 m	5	9
SIB	Seonginbong, Ulleung Island	37°30′07.9′′N 130°52′13.2′′E	904 m	8	19
THR	Taeha-ri, Ulleung Island	37°29′46.52′′N, 130°48′45.71′′E	222 m	5	10
Others**	Dodong-ri and Bonghwa Falls, Ulleung Island	–	–		3
*Prunus sargentii* Rehder					
JJ**	Jeju Island, South Korea			7	10
ODS	Odaesan, South Korea	37°48′10.5′′N 128°33′59.2′′E	1282 m	5	11
RSS	Primorskiy Krai, Chekhovo, and Sakhalin Oblast, Russia**			5	4
MYG	Miyagi, Japan	38°19′31.2′′N 140°37′00.6′′E	302 m	8	12
OKA	Okayama, Japan	34°52′28.0′′N 133°39′43.4′′E	347 m	5	4
Others**	Mt. Jiri (JRS), Sobaek (SBK), and Hambaek (HBK) in the Korean Peninsula	–	–	–	5

N/I, number of individuals in population. *Same locality as NAR, but individuals with hairs on pedicel. Population excluded from cpDNA network analysis. **Samples were obtained from multiple spots within specified localities, thus exact GPS data are not provided.

### Genotyping of MIG-seq SNPs

The MIG-seq library was constructed by two-step amplification, as detailed by Suyama and Matsuki (2015), using 288 samples representing 14 *Cerasus* species, including *P. takesimensis* and *P. sargentii*. The first PCR was performed from genomic DNA using designated primers to target ISSR, followed by a second PCR to add the complementary sequences for the binding sites of the Illumina sequencing flow cell and indices (barcodes) as specified by Suyama and Matsuki (2015) to the first PCR amplicons. After purification, the amplified products were pooled and sequenced on an Illumina MiSeq Sequencer (Illumina, San Diego, CA, United States) for the selected sizes of 350–800 base pairs (bp) using the MiSeq Reagent Kit v3 (150 cycles PE, Ref. 15043893). Low-quality ends of reads, SSR primer regions, anchors, and index-tags were removed from the obtained NGS data using the FASTX Toolkit^1^ and TagDust 1.12 (Lassmann et al., 2009). A total of 26 samples with extensive missing data were removed after checking by vcftools (Danecek et al., 2011) to maintain the optimal dataset quality. The processed reads were analyzed to discover SNPs using STACKS v1.48 (Catchen et al., 2013), generating output data matrices in multiple formats of STRUCTURE, Phylip, and variant call format (VCF) for phylogenetic and population genetic analyses. In STACKS, we used the program ‘ustacks’ to assemble and pile stacks *de novo* using the default value of the minimum depth of coverage required (-m 3) and the maximum distance allowed between stacks (-M 2). Deleveraging (d) and removal (r) algorithms were enabled. The program ‘cstacks’ was used to create a catalog, and ‘ustacks’ products were searched against the catalog in the program ‘sstacks.’ The SNP data matrix, including 262 samples, was generated by the program ‘populations’ using the optimized parameters based on the most robustness in phylogenetic analysis after testing variable parameters (–*r* = 0.5, 0.75; –*p* = 2, 8, 16, 24, 32, and 40) as shown in Supplementary Table 2. The applied parameters were; at least 75% (–r 0.75) of minimum percentage of individuals across populations, minimum eight populations present to process a locus (–p 8), minimum minor allele frequency at a locus of 0.05 (–min-max 0.05), and maximum observed heterozygosity at a locus of 0.95 (–max-obs-het 0.95).

### Phylogenetic Analysis, Network Construction, and Population Structure

To determine the phylogenetic position of *P. takesimensis* among other *Cerasus* species, we first conducted maximum likelihood (ML) analyses of the nrDNA ITS/ETS and the concatenated seven non-coding regions of the cpDNA datasets for 123 samples using W-IQ-TREE (Trifinopoulos et al., 2016), an intuitive and user-friendly web interface and server for IQ-TREE (Nguyen et al., 2015). An IQ-TREE was also constructed from genome-wide 5899 SNPs in the MIG-seq dataset for 262 *Cerasus* samples using *P. maximowiczii* Ruprecht from the section *Phyllomahaleb* Koehne as an outgroup taxon. Ultrafast bootstrap support (BS) was calculated from 1000 bootstrap replicates for the robustness of the clades (Hoang et al., 2018). Best-fit substitution models were determined according to the Bayesian information criterion using ModelFinder (Kalyaanamoorthy et al., 2017) implemented in IQ-TREE: HKY + F + I for nrDNA ITS/ETS, F81 + F + I + G4 for cpDNA seven non-coding regions, and TVM + F + ASC + G4 for MIG-seq dataset. Additionally, a SVDQuartets bootstrap consensus tree (Kubatko and Degnan, 2007) was generated based on MIG-seq dataset partitioned for species and populations (for *P. takesimensis* and *P. sargentii*) from default setting of 100,000 random quartets with QFM quartet tree search algorithm and bootstrapping of 100 replicates in PAUP 4.0a169 (Swofford, 2020).

For the population-level analysis of insular endemic *P. takesimensis* and the purported continental progenitor *P. sargentii*, we constructed a cpDNA haplotype network using TCS version 1.21 (Clement et al., 2000) from five concatenated cpDNA datasets for 99 accessions of both species (Table 1). Gaps were treated as missing data, and the probability of parsimony was set to 95% of the connection limit in accordance with Hart and Sunday (2007). Using the same dataset, genetic variation between both species, and among and within populations was evaluated by analysis of molecular variance (AMOVA) using ARLEQUIN ver. 3.5 (Excoffier et al., 2005).

The genetic diversity and structure were also analyzed using the MIG-seq dataset based on genome-wide SNPs. Population genetic structure was estimated by the Bayesian model-based genetic clustering method in STRUCTURE ver. 2.3 (Pritchard et al., 2000) based on SNP matrices generated in STACKS using “–write_single_snp” command for avoiding the potential bias from linked SNPs in the same loci. The STRUCTURE computation was run for both datasets; i.e., the population-based dataset of *P. takesimensis* (14 populations) and *P. sargentii* (5 populations), including 200 samples (see Table 1 for population information excluding minor samples from various localities), and the dataset including a total of 262 samples of all *Cerasus* species used in MIG-seq analysis (Supplementary Table 1). The optimal *K* value for each analysis was estimated by the maximum value of ΔK following the Evanno method (Evanno et al., 2005), implemented in STRUCTURE HARVESTER (Earl and vonHolt, 2012). Genetic diversity in the populations of both species was measured using the program ‘populations’ in STACKS v1.48 (Catchen et al., 2013). To examine genetic similarities and relationships between individuals, we conducted a principal component analysis (PCA) using the glPCA command of R statistical software, R 4.0.2, in RStudio based on the STACKS output file in VCF format from the MIG-seq analysis of 262 *Cerasus* samples.

## Results

### Phylogenetic Tree Derived From SNPs of MIG-seq Analysis

**FIGURE 2:**
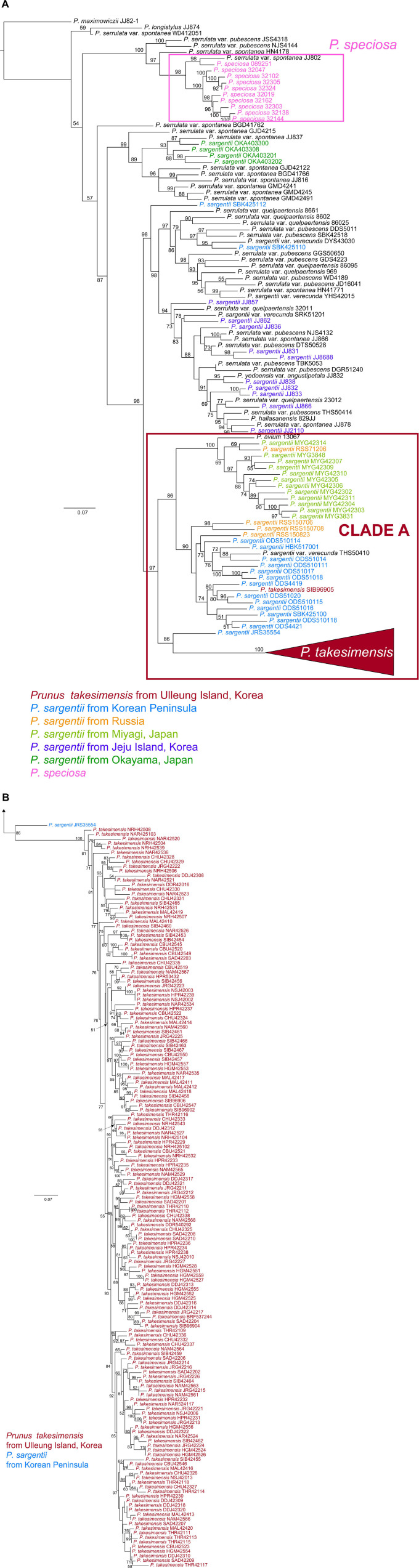
Maximum likelihood (ML) tree produced by IQ-TREE based on SNPs detected by MIG-seq analysis, including 262 accessions of flowering cherries of the subgenus *Cerasus* (the genus *Prunus*). Numbers above branches indicate bootstrap support (BS) percentages of > 50%. **(A)** In total, 161 accessions of *P. takesimensis* are collapsed in the red triangle. Color codes for species and geographical regions: red for *P. takesimensis* from Ulleung Island, blue for *P. sargentii* from Korean Peninsula, orange for *P. sargentii* from Russia, light green for *P. sargentii* from Miyagi, Japan, purple for *P. sargentii* from Jeju Island, South Korea, dark green for *P. sargentii* from Okayama, Japan, and pink for *P. speciosa*. **(B)** The part of ML tree for collapsed accessions of *P. takesimensis* in the red triangle.

Phylogenetic analyses of 123 accessions of *Cerasus* species did not resolve the phylogenetic relationship between *P. takesimensis* and *P. sargentii* because of the lack of resolution in ML tree of nrDNA ITS and ETS (956 aligned sites; see data matrices available in dryad under doi: 10.5061/dryad.x3ffbg7jn) (Supplementary Figure 1) and non-monophyly of both species in the seven concatenated cpDNA ML tree (5414 aligned sites; see data matrices available in dryad under doi: 10.5061/dryad.x3ffbg7jn) (Supplementary Figure 2). It was apparent that all but one species, *P. maximowiczii* (section *Phyllomahaleb*), were poorly resolved with low nodal supports. However, MIG-seq phylogeny based on genome-wide SNPs (262 accessions and 5899 total SNPs; see data matrices available in dryad under doi: 10.5061/dryad.x3ffbg7jn) provided much greater resolution (Figures 2A,B). Specifically, two endemic flowering cherry species with relatively narrow distribution, *P. takesimensis* on Ulleung Island, South Korea, and *P. speciosa* on Izu Islands and Izu Peninsula, Japan, were primarily monophyletic, although several other *Cerasus* species remain unresolved. For the first time, the species boundary of *P. takesimensis* was delimited at the molecular level, and more importantly, its origin could be assessed through the relationship between continental progenitors and insular derivative species suggested by the MIG-seq phylogeny. *P. takesimensis* was nested within one lineage (CLADE A; 97% BS value) of its purported continental progenitor species *P. sargentii*, mainly sampled from populations of the Korean Peninsula, Russia, and Miyagi, Japan. *P. takesimensis* was found to be monophyletic; a total of 162 accessions formed a highly supported monophyletic group (100% BS value) except for one accession (SIB969-005), which was also nested in the group of *P. sargentii* accessions collected from Mt. Odaesan in the central part of the Korean Peninsula. The clade comprising all but one accession of *P. takesimensis* shared the most recent common ancestor with *P. sargentii* JRS355-54 collected from Mt. Jirisan in the southern part of the Korean Peninsula (Figure 2A). Within *P. takesimensis* on Ulleung Island, no geographical patterns were observed, including the hairy population (NRH) (Figure 2B). Contrary to *P. takesimensis*, the purported continental progenitor *P. sargentii* was not monophyletic; accessions collected from Jeju Island and Okayama, Japan were closely related to *P. serrulata* species. However, the topology of the MIG-seq phylogenetic tree revealed the geographic structure of *P. sargentii* (Figure 2A). Within CLADE A, the accessions of *P. sargentii* collected from geographically connected continental areas of the Korean Peninsula and Russia clustered together, except for one accession (*P. sargentii* RSS71206 was nested in the Miyagi population). In contrast, the accessions of *P. sargentii* collected from Okayama, Japan were distantly related to *P. takesimensis* and were embedded within the lineage of *P. serrulata var. spontanea*, comprising the accessions collected from the southern islands of South Korea (i.e., Geoje Island, Geomun Island, Bogil Island, and Jeju Island). *P. sargentii* accessions collected from Jeju Island were nested in the group, which included other *Cerasus* species from Jeju Island and the Korean Peninsula and was in the sister relationship to CLADE A (Figure 2A).

**FIGURE 3:**
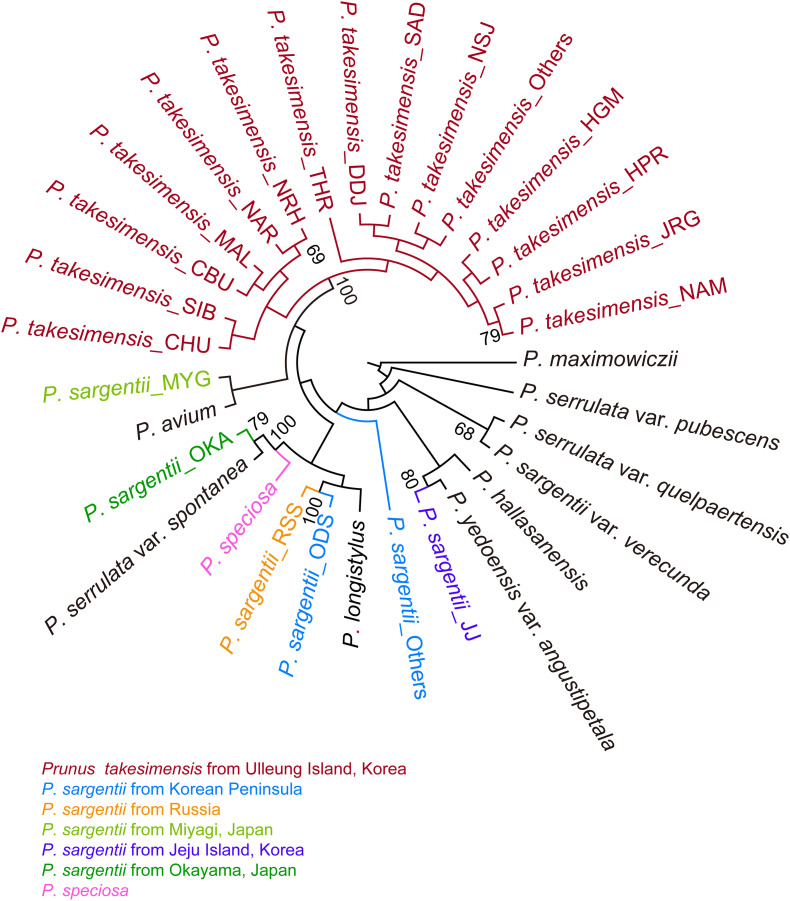
SVDQuartets bootstrap consensus tree produced by PAUP based on MIG-seq dataset including 262 accessions of flowering cherries of the subgenus *Cerasus* (the genus *Prunus*) partitioned for species and populations for *P. takesimensis* and *P. sargentii*. Population codes are shown in Table 1. Color codes for species and geographical regions: red for *P. takesimensis* from Ulleung Island, blue for *P. sargentii* from Korean Peninsula, orange for *P. sargentii* from Russia, light green for *P. sargentii* from Miyagi, Japan, purple for *P. sargentii* from Jeju Island, South Korea, dark green for *P. sargentii* from Okayama, Japan, and pink for *P. speciosa*. Numbers above major branches indicate bootstrap support (BS) percentages.

The SVDQuartets bootstrap consensus tree reconfirmed strongly the monophyly of *P. takesimensis* (100% BS). However, the geographic origin of *P. takesimensis* could not be inferred from SVDQuartets analysis, as other clades resolved in ML tree were highly unresolved without providing any clue to the origin of *P. takesimensis*. Clade A in ML tree, including *P. takesimensis* and the populations of the Korean Peninsula, Russia, and Miyagi, Japan of *P. sargentii*, was not resolved in the SVDQuartets bootstrap consensus tree. *Prunus takesimensis* shared the most recent common ancestor with the lineage comprising *P. sargentii* and *P. avium* collected from Miyagi, Japan, but with very weak support value (<50% BS) (Figure 3).

### cpDNA Haplotype Network and Relationship Between *P. takesimensis* and *P. sargentii*

**FIGURE 4:**
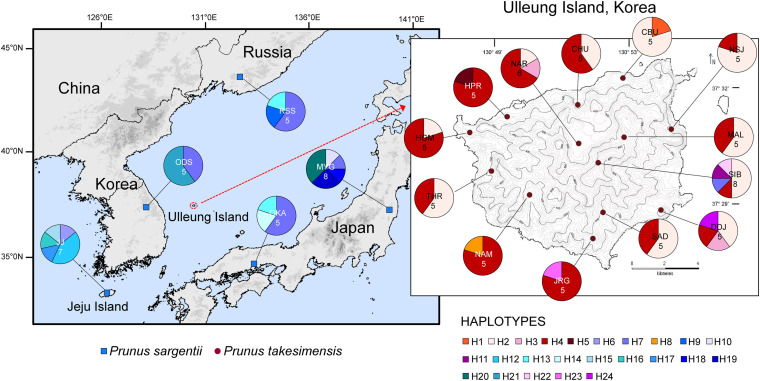
Map of the haplotypes found in the populations of the insular derivative, *P. takesimensis*, and its continental progenitor species, *P. sargentii*, based on five non-coding regions of cpDNA. Populations are labeled with different color codes specified in Table 1. The sampling locations are denoted in different colors: red for *P. takesimensis* and blue for *P. sargentii.* Different colored portions in each pie chart represent the haplotype frequencies.

The aligned sequence length of five concatenated chloroplast non-coding regions used for the construction of the TCS haplotype network was 3,809 characters (see data matrices available in dryad under doi: 10.5061/dryad.x3ffbg7jn): *pet*A-*psb*J (1–931; 931 sites), *pet*D-*rpo*A (932–1,222; 291 sites), *rpl*16 intron (1,223–2,076: 854 sites), *trn*L-*rpl*32 (2,077–3,217; 1,141 sites), and *trn*Q-*rps*16 (3,218–3,809; 592 sites). The TCS haplotype network contained 24 haplotypes in total: 11 haplotypes for *P. takesimensis* (69 accessions from 13 populations) and 14 haplotypes for *P. sargentii* (30 accessions from five populations). Of the 24 haplotypes, 10 were exclusive to *P. takesimensis*, 13 to *P. sargentii*, and only one (H7) was shared by both species in nine accessions of *P. sargentii* and one accession of *P. takesimensis* (SIB2115-6) as shown in Figure 4. The number of haplotypes and polymorphic sites within each population is specified in Table 2. Given the number of populations and haplotypes within each population, the diversity of haplotypes was markedly higher in the populations of *P. sargentii* than in *P. takesimensis*, despite the considerably broad sampling of *P. takesimensis*. The number of haplotypes within populations of *P. sargentii* ranged from two (ODS) to five (JJ and MYG) with higher gene diversity and nucleotide diversity (mean 0.7428 and 0.0081, respectively) than in *P. takesimensis* (mean 0.5451 and 0.0049, respectively). Most populations of *P. takesimensis* showed a simple combination of only two haplotypes, except NAR (three haplotypes), DDJ (four haplotypes), and SIB (five haplotypes).

**TABLE 2:** Distribution of haplotypes (frequency in parentheses) and genetic diversity among the populations of the insular derivative, *P. takesimensis*, and continental progenitor species, *P. sargentii*, based on five non-coding regions of cpDNA.

Species	Population Code	N/I	Haplotype (Frequency)	No. of polymorphic sites	Gene diversity	Nucleotide diversity
*Prunus takesimensis*	CBU	5	H1 (1)	13	0.4000 ± 0.2373	0.001400 ± 0.000953
			H2 (4)			
	CHU	5	H2 (2)	41	0.6000 ± 0.1753	0.006616 ± 0.004116
			H4 (3)			
	DDJ	5	H2 (2)	47	0.9000 ± 0.1610	0.007152 ± 0.004441
			H3 (1)			
			H4 (1)			
			H24 (1)			
	HGM	5	H2 (1)	41	0.4000 ± 0.2373	0.004411 ± 0.002780
			H4 (4)			
	HPR	5	H4 (4)	1	0.4000 ± 0.2373	0.000109 ± 0.000138
			H5 (1)			
	JRG	5	H4 (4)	15	0.4000 ± 0.2373	0.001629 ± 0.001093
			H23 (1)			
	MAL	5	H2 (3)	41	0.6000 ± 0.1753	0.006616 ± 0.004116
			H4 (2)			
	NAM	5	H4 (4)	29	0.4000 ± 0.2373	0.003130 ± 0.002004
			H8 (1)			
	NAR	6	H2 (1)	41	0.6000 ± 0.2152	0.004483 ± 0.002695
			H3 (1)			
			H4 (4)			
	NSJ	5	H2 (4)	41	0.4000 ± 0.2373	0.004411 ± 0.002780
			H4 (1)			
	SAD	5	H2 (3)	41	0.6000 ± 0.1753	0.006616 ± 0.004116
			H4 (2)			
	SIB	8	H2 (4)	101	0.7857 ± 0.1508	0.010525 ± 0.005848
			H4 (1)			
			H7 (1)			
			H11 (1)			
			H22 (1)			
	THR	5	H2 (3)	41	0.6000 ± 0.1753	0.006616 ± 0.004116
			H4 (2)			
	Total	69	11 haplotypes	Mean 37.923	Mean 0.5451	Mean 0.0049
			(10 exclusive)			
*Prunus sargentii*	JJ	7	H6 (1)	25	0.8571 ± 0.1371	0.002190 ± 0.001327
			H12 (3)			
			H15 (1)			
			H16 (1)			
			H17 (1)			
	ODS	5	H7 (2)	49	0.6000 ± 0.1753	0.008110 ± 0.005024
			H21 (3)			
	RSS	5	H7 (3)	52	0.7000 ± 0.2184	0.008711 ± 0.005396
			H9 (1)			
			H13 (1)			
	MYG	8	H7 (1)	99	0.8571 ± 0.1083	0.013708 ± 0.007585
			H10 (1)			
			H18 (1)			
			H19 (2)			
			H20 (3)			
	OKA	5	H7 (3)	49	0.7000 ± 0.2184	0.007945 ± 0.004923
			H13 (1)			
			H14 (1)			
	Total	30	14 haplotypes	Mean 54.8	Mean 0.7428	Mean 0.0081
			(13 exclusive)			

*Population code: please refer to Table 1 for exact localities of each population. N/I, number of individuals sampled in this population at this site.*

**FIGURE 5:**
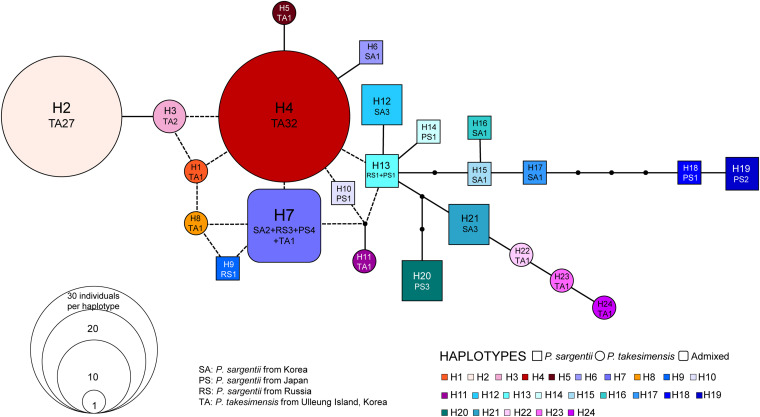
TCS haplotype network constructed from five non-coding regions of cpDNA. Relationships among the 24 haplotypes found in *P. takesimensis* on Ulleung Island (circles) and its continental progenitor, *P. sargentii*, from adjacent areas (squares). Small dots represent either missing or inferred haplotypes and the size of each circle and square is proportional to the population size.

The genealogical relationships among cpDNA haplotypes are shown in Figure 5. In general, the haplotypes of *P. takesimensis* and *P. sargentii* were separated from each other in the network; however, the distinction between both species was not complete, with the exception of some haplotypes. Haplotype H7 was found primarily in *P. sargentii* (nine accessions), with one accession from *P. takesimensis* forming a ring-like network structure with other closely related haplotypes H4, H8, and H9. Haplotype H11 was found only in a single accession of *P. takesimensis* (SIB2115-1) and was derived by one mutational step from a missing haplotype of another ring structure composed of *P. sargentii* haplotypes H13, H10, and H7. Three haplotypes of *P. takesimensis*, H22 (SIB population), H23 (JRG population), and H24 (DDJ population), were derived from H21 exclusively found in *P. sargentii* (ODS). The two dominant haplotypes found in the populations of *P. takesimensis* were H4 (shared by 32 accessions) and H2 (shared by 27 accessions), while the other haplotypes were represented by only a single accession (seven haplotypes; H1, H5, H8, H11, H22, H23, and H24) or two (one haplotype; H3). In contrast, no dominant haplotypes were found in *P. sargentii* populations. With regard to the partitioning of genetic variation, including both species of *P. sargentii* and *P. takesimensis*, the majority of variation (59%) existed within populations, while approximately 30% and 10% of the variation existed between species and among populations within species, respectively (Table 3). Within the continental progenitor species, *P. sargentii*, the majority of the variation (86.4%) occurred within populations, while the remaining variation (13.6%) existed among populations. A similar level of genetic variation was found within populations (83.7%) for the insular derivative species *P. takesimensis*.

**TABLE 3:** Summary of the analysis of molecular variance (AMOVA) found between *P. takesimensis* and *P. sargentii* based on five non-coding regions of cpDNA.

Species	Source of variation	d.f.	Sum of squares	Variance components	Percentage variation (%)	Fixation indices	*P*-value
*P. sargentii*,	Between species	1	270.822	5.92644	30.95	*F*_*CT*_: 0.30947	0.00000 ± 0.00000
*P. takesimensis*	Among populations within species	16	350.422	1.94731	10.17	*F*_*SC*_: 0.14726	0.00684 ± 0.00231
	Within populations	81	913.402	11.27657	58.88	*F*_*ST*_: 0.41115	0.00000 ± 0.00000
	Total	98	1534.646	19.15032			
*P. sargentii*	Among populations	4	115.823	2.35470	13.58	*F*_*ST*_: 0.13580	0.06549 ± 0.00890
	Within populations	25	374.611	14.98443	86.42		
	Total	29	490.433	17.33913			
*P. takesimensis*	Among populations	12	234.600	1.87436	16.30	*F*_*ST*_: 0.16305	0.03128 ± 0.00455
	Within populations	56	538.792	9.62128	83.70		
	Total	68	773.391	11.49564			

*d.f., degrees of freedom; F_CT_, proportion of genetic variation between species; F_SC_, proportion of genetic variation among populations within species; F_ST_, proportion of genetic variation among all populations and species overall.*

### Genetic Diversity and Population Structure From MIG-seq Analysis

**FIGURE 6:**
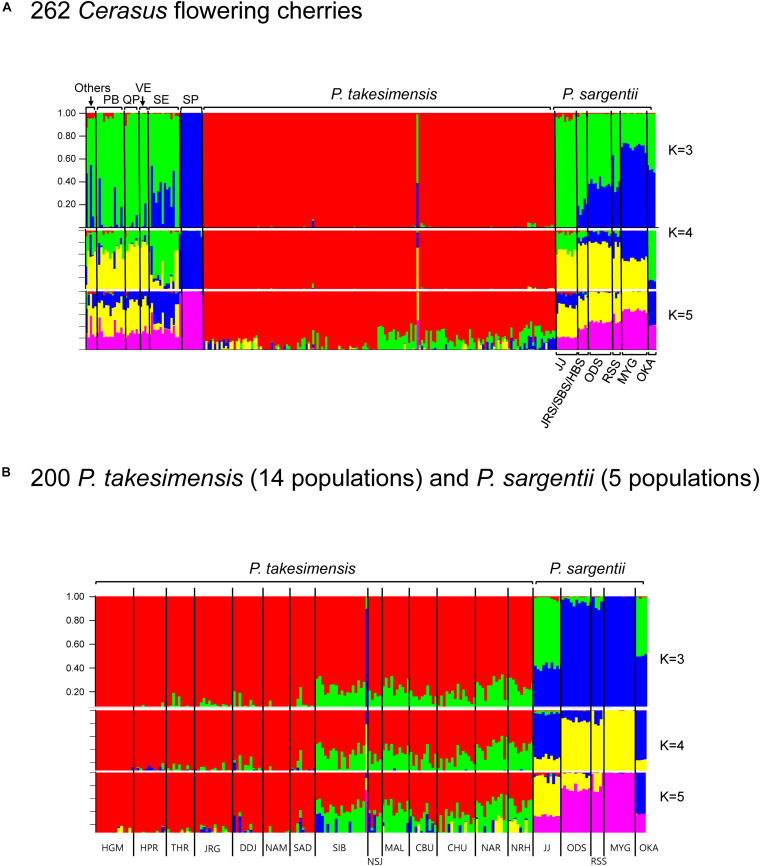
Population genetic structure by Bayesian clustering using STRUCTURE based on MIG-seq SNPs. Bar plots illustrate the population structure at *K* = 3, 4, and 5 in 262 *Cerasus* flowering cherries **(A)**, and 200 of *P. takesimensis* (14 populations) and *P. sargentii* (5 populations) **(B)**. Each individual is represented by a single vertical line composed of *K* colored segments, with lengths proportional to estimated membership percentage of the *K* clusters, respectively. Bar plot for best *K* value (*K* = 3 for both 262 and 200 datasets) determined by STRUCTURE HARVESTER is depicted larger than the bar plots of other Ks. Species name abbreviations: PB for *P. serrulata* var. *pubescens*, QP for *P. serrulata* var. *quelpaertensis*, VE for *P. sargentii* var. *verecunda*, SE for *P. serrulata* var. *spontanea*, and SP for *P. speciosa*. *“*Others” includes the species of *P. yedoensis* var. *angustipetala*, *P*. *avium*, *P. longistylus*, *P. hallasanensis*, and *P. maximowiczii.* For population codes, please refer to Table 1.

Based on SNP matrices generated by STACKS, the genetic structure was estimated first for 262 accessions of all *Cerasus* species (8527 loci, see data matrices available in dryad under doi: 10.5061/dryad.x3ffbg7jn) using STRUCTURE 2.3.4 (Pritchard et al., 2000; Figure 6A). Further comparison between insular endemic species and purported continental progenitor species was made for 200 accessions, including 14 populations of *P. takesimensis* and five populations of *P. sargentii* (7038 loci, see data matrices available in dryad under doi: 10.5061/dryad.x3ffbg7jn) (Figure 6B). The best *K* value was identified as three clusters (*K* = 3) for both datasets based on the rate of change in the log probability of data between successive *K* values (Evanno et al., 2005) from STRUCTURE HARVESTER (Earl and vonHolt, 2012). The summary of partitioned *K* = 3 bar plots for total *Cerasus* species showed that the two endemic flowering cherry species of *P. takesimensis* on Ulleung Island, South Korea and *P. speciosa* on Izu Islands and Izu Peninsula, Japan, were distinct from other *Cerasus* cherry species in the composition of genetic clusters (Figure 6A). Specifically, *P. takesimensis* was differentiated from the purported continental progenitor species, *P. sargentii*. It was represented by its own exclusive single cluster (with the lowest heterozygosity, 0.2119), while *P. sargentii* was represented by different genetic clusters (with relatively higher heterozygosity, 0.2556 and 0.2844) shared with other *Cerasus* species with admixed (or single) composition. One accession of *P. takesimensis* (SIB969-005) appeared as an outlier, showing genetic profiles similar to those of *P. sargentii* from ODS and RSS population. All accessions of *P. speciosa* were extremely uniform in its genetic composition comprising single cluster (heterozygosity; 0.2556) from all K values. The populations of *P. sargentii* differed in genetic profiles; Jeju and Okayama populations showed admixed profiles similar to those of *P. serrulata* var. *quelpaertensis* and *P. serrulata* var. *spontaneae*, and were differentiated from three populations of Odaesan in the Korean Peninsula, Russia, and Miyagi, Japan. The results of comparison between both species, *P. takesimensis* and *P. sargentii*, also revealed clear differentiation in population genetic structures, as displayed in the bar plots of all *K* values (Figure 6B). There were very weak genetic structures among populations of *P. takesimensis* geographically for *K* = 3 and 4 analyses, with a little bit stronger geographic patterns between the southwestern and northeastern parts of the island with *K* = 5 analysis. The southwestern populations (HGM, HPR, THR, JRG, NAM, SAD, SIB, and DDJ) and northeastern populations (NSJ, MAL, CBU, CHU, NAR, and NRH) slightly differed in the proportions of the inferred clusters. One outlier accession (SIB969-005) was similar to *P. sargentii* from the ODS, RSS, and MYG populations.

PCA results based on 262 MIG-seq dataset in VCF format (see data matrices available in dryad under doi: 10.5061/dryad.x3ffbg7jn) further confirmed the genetic relationship among accessions of *Cerasus* species. *P. takesimensis* and *P. speciosa* were distinct from other species in the PCA scatter plot constructed by PC1 and PC2 (Supplementary Figure 3). The other species overlapped on both the PC1 and PC2 axes and were not separated from each other. Genetic diversity was estimated from the 200 accession SNP dataset of *P. takesimensis* (14 populations, 159 accessions) and *P. sargentii* (five populations, 41 accessions) using STACKS. The expected and observed heterozygosity were higher in the populations of *P. takesimensis* (mean values of 0.1543 and 0.1905) than in *P. sargentii* (mean 0.1241 and 0.1658) (Table 4).

**TABLE 4:** Molecular diversity indices estimated in the populations of the insular derivative, *P. takesimensis*, and continental progenitor species, *P. sargentii*, based on MIG-seq SNPs.

Species	Code	N/I	N	Private	P	H_*E*_ (o)	H_*O*_ (o)	H_*E*_ (e)	H_*O*_ (e)	π	F_*IS*_
*P. takesimensis*	CBU	10	9.3391	0	0.8601	0.1658	0.8342	0.2006	0.7994	0.2121	0.1286
	CHU	14	12.875	0	0.8606	0.1585	0.8415	0.201	0.799	0.2093	0.1545
	DDJ	12	10.7656	0	0.8687	0.1555	0.8445	0.1871	0.8129	0.1964	0.1205
	HGM	14	12.7902	0	0.8769	0.151	0.849	0.1742	0.8258	0.1813	0.0909
	HPR	12	10.7659	0	0.8635	0.1407	0.8593	0.1908	0.8092	0.2003	0.1694
	JRG	14	12.688	0	0.8658	0.1546	0.8454	0.1938	0.8062	0.2019	0.1434
	MAL	10	9.3234	0	0.8641	0.1583	0.8417	0.1945	0.8055	0.2057	0.1293
	NAM	9	8.1962	0	0.8748	0.1433	0.8567	0.1793	0.8207	0.1912	0.1342
	NAR	11	10.1103	0	0.855	0.1598	0.8402	0.2052	0.7948	0.2161	0.1688
	NRH	10	9.2986	0	0.8653	0.1621	0.8379	0.1928	0.8072	0.2038	0.1194
	NSJ	5	4.5185	0	0.8725	0.1453	0.8547	0.1757	0.8243	0.1983	0.1151
	SAD	9	8.242	0	0.8703	0.1564	0.8436	0.1837	0.8163	0.1959	0.1023
	SIB	19	17.1147	2	0.8541	0.1559	0.8441	0.2092	0.7908	0.2156	0.2324
	THR	10	9.2161	0	0.8749	0.153	0.847	0.1785	0.8215	0.1889	0.1019
**Mean**			**10.3745**		**0.8662**	**0.1543**	**0.8457**	**0.1905**	**0.8095**	**0.2012**	**0.1365**
*P. sargentii*	JJ	10	9.0748	4	0.8389	0.1565	0.8435	0.22	0.78	0.233	0.1975
	MYG	12	10.7478	6	0.8776	0.1202	0.8798	0.1646	0.8354	0.1728	0.1317
	OKA	4	3.6493	3	0.9284	0.0775	0.9225	0.0947	0.9053	0.1104	0.0629
	ODS	11	10.0497	1	0.866	0.1393	0.8607	0.1839	0.8161	0.1936	0.1448
	RSS	4	3.5507	0	0.8769	0.127	0.873	0.1657	0.8343	0.1937	0.1294
**Mean**			**7.4145**		**0.8776**	**0.1241**	**0.8759**	**0.1658**	**0.8342**	**0.1807**	**0.1333**

*Code: Population code (Please refer to Table 1 for exact localities of each population). N/I, number of individuals sampled in this population at this site. N, mean number of individuals per locus in this population. Private: Number of private alleles in this population. P, mean frequency of the most frequent allele at each locus in this population. HE (o), mean observed heterozygosity (The proportion of individuals that are heterozygotes in this population). HE (e), mean expected heterozygosity under Hardy-Weinberg equilibrium in this population. HO (o), mean observed homozygosity (The proportion of individuals that are homozygotes in this population). HO (e), mean expected homozygosity under Hardy-Weinberg equilibrium in this population. π, mean value of π (an estimate of nucleotide diversity) in this population. FIS, mean measure of FIS (value of inbreeding coefficient) in this population. HO, HE, π, and FIS were calculated at variant nucleotide sites. The mean values are bolded for comparison between both species of P. takesimensis and P. sargentii.*

## Discussion

### Taxonomic Distinction and Origin of Ulleung Island Flowering Cherry

In this study, our first aim was to examine the taxonomic identity of *P. takesimensis* as it diverged from its presumptive continental progenitor, *P. sargentii*, as well as other flowering cherry species. Morphologically, *P. takesimensis* has been recognized as a distinct species from other flowering cherries occurring in adjacent continents and other islands (Chang et al., 2004). However, its monophyly and taxonomic distinction have never been confirmed in previous phylogenetic analyses. *Cerasus* species of flowering cherries are often delimitated based on several diagnostic features (e.g., inflorescence type, degree of pubescence on flowers and leaves, and phenology), but such morphological delineation has not been supported by genetic distinction in molecular phylogenetic analyses. For example, the *P. serrulata*/*P. sargentii* complex (Cho et al., 2014; Cho and Kim, 2019), which provides a clue regarding the origin of *P. takesimensis*, has been shown to be non-monophyletic because of insufficient sequence variation in Sanger sequencing-based approaches, lineage sorting, introgression, and hybridization. Therefore, the phylogenetic relationship among *Cerasus* species could not be inferred in previous studies, which hindered identifying the origin and evolution of *P. takesimensis*, as well as its relationship with the purported continental progenitor, *P. sargentii*.

To the best of our knowledge, this is the first study to provide convincing evidence regarding the monophyly of *P. takesimensis* and identify it as a discrete taxonomic entity on Ulleung Island, South Korea. Its taxonomic distinction and phylogenetic position were established using multiple lines of evidence, including cpDNA haplotype network analysis and robust phylogenetic and population genetic analyses using genome-wide MIG-seq SNPs. Based on the haplotype network, *P. takesimensis* (insular derivative) was differentiated from *P. sargentii* (purported continental progenitor), as the former possessed species diagnostic haplotypes exclusively (with one exceptional haplotype H7) (Figures 4, 5). However, the haplotype relationships did not provide additional phylogenetic inferences into species relationships. The MIG-seq SNP data provided well-resolved phylogenetic relationships and strongly supported the monophyly of *P. takesimensis*. In both of the ML tree and SVDQuartets bootstrap consensus tree, *P. takesimensis*, with the exception of one outlier (in ML tree), formed a 100% BS-supported monophyletic group, suggesting its genetic cohesiveness on Ulleung Island (Figures 2A, 3). The genetic differentiation of *P. takesimensis* from its purported continental progenitor, *P. sargentii*, as well as other flowering cherries, was further identified unambiguously by genetic structure and PCA analyses; *P. takesimensis* shows unique genomic profiles that are strongly differentiated from other *Cerasus* species (Figure 6 and Supplementary Figure 3).

In addition to the monophyly of *P. takesimensis*, MIG-seq SNP phylogenetic reconstruction revealed the continental progenitor and insular derivative species relationship between *P. sargentii* and *P. takesimensis*, which provided clues to the origin of *P. takesimensis*. The MIG-seq method successfully uncovered the species delimitation and phylogenetic relationships of the genetic lineages confined to relatively narrow (or isolated) distribution areas, such as *P. speciosa* (endemic to the Izu Islands and Izu Peninsula, Japan) and *P. takesimensis* on Ulleung Island. Furthermore, three geographic groups of *P. sargentii* are recognized in the MIG-seq ML phylogeny, that is, OKA (Okayama, Japan), JJ (Jeju Island in South Korea), and the combined clade (Clade A) of MYG (Miyagi, Japan), ODS/SBK/HBK/JRS (the Korean Peninsula) and RSS (Russia), which facilitates tracing the geographic origin of the continental progenitor of *P. takesimensis*. The strongest phylogenetic evidence for detecting progenitor-derivative species pairs comes from insular endemic populations of one species (derivative) nested within source populations of another species (progenitor) (Crawford, 2010). In the ML tree constructed by IQ-TREE, all accessions of *P. takesimensis* were nested within the populations of *P. sargentii* (Clade A), and we could reconfirm the continental progenitor and insular derivative species relationship between *P. sargentii* and *P. takesimensis*, which was initially presumed based on morphological similarities. All but one accession of *P. takesimensis* (161 accessions) formed a monophyletic group (100% BS), sharing the most recent common ancestor with one accession (JRS35554) of *P. sargentii* collected from Mt. Jirisan in the southern part of the Korean Peninsula (86% BS) (Figure 2A). This suggests that *P. takesimensis* could originate from a single colonization event, most likely from the continental progenitor of *P. sargentii* from the Korean Peninsula. Given the abundance and wide range of *P. takesimensis* on the island, a single origin has not been expected due to the geographical proximity of Ulleung Island from adjacent possible source areas. However, the single origin of *P. takesimensis* is concordant with other anagenetically derived endemic species on Ulleung Island, such as *Acer takesimense* (Sapindaceae; Takayama et al., 2013), *Acer okamotoanum* (Sapindaceae; Takayama et al., 2012), *Campanula takesimana* (Campanulaceae; Cheong et al., 2020), *Dystaenia takesimana* (Apiaceae; Pfosser et al., 2005), and *Phedimus takesimensis* (Crassulaceae; Seo et al., 2020). Currently, a single origin is considered to be the norm for anagenetically originated endemic plants on Ulleung Island (Seo et al., 2020), including *P. takesimensis* in the present study, although there are few exceptions with multiple origins of other endemic species, such as *Rubus takesimensis* (Rosaceae; Yang et al., 2019) and *Scrophularia takesimensis* (Scrophulariaceae; Gil et al., 2020). Therefore, *P. takesimensis* represents an additional endemic taxon anagenetically derived on Ulleung Island and evolved by a single introduction highly likely from a source population of *P. sargentii* on the southern part of the Korean Peninsula according to the ML phylogeny.

However, the origin of *P. takesimensis* seems still uncertain, although a single origin of *P. takesimensis* from the *P. sargentii* population in Korean Peninsula is highly plausible (Figure 2A). SVDQuartets analysis found that high proportion of quartets (ca. 43%) are incomparable with the tree, which would indicate incomplete lineage sorting and other processes, such as introgression or paralogous sequences in the alignment. Frequent hybridization and introgression among congeneric species of flowering cherries have been documented in previous studies, making the species relationships rather uncertain (Bortiri et al., 2001, 2002; Ohta et al., 2007; Potter, 2011; Cho et al., 2014; Cho and Kim, 2019). Unlike the ML phylogeny, SVDQuartets analysis did not provide the decisive clue to the origin of *P. takesimensis*. *Prunus takesimensis* shared the most recent common ancestor with *P. avium* and *P. sargentii* from Miyagi, Japan, which was, however, very weakly supported (<50% BS) (Figure 3). In addition, depending on the number of SNPs generated by different parameter settings in STACKS analysis (ML trees not shown), the phylogenetic position of *P. takesimensis* differed slightly relative to source populations of *P. sargentii* within Clade A. Furthermore, we cannot rule out the possibility of multiple potential origins, taking into account the genetic and phenotypic variations found in the populations of *P. takesimensis*. One accession of *P. takesimensis* (SIB969-005) was nested within the ODS population of *P. sargentii* (Mt. Odaesan in the central part of the Korean Peninsula) instead of being nested within the conspecific monophyletic group on MIG-seq phylogeny. Another accession of *P. takesimensis* (SIB2115-6), which was not included in the MIG-seq analysis, shared the same haplotype (H7) as *P. sargentii* accessions. These two genetic outliers within the same population (SIB; Mt. Seonginbong, Ulleung Island) potentially represent independent dispersal event and convergent or parallel evolution.

While the evidence for monophyly of *P. takesimensis* (100% BS) and identification of the clade containing *P. sargentii* populations from mainland South Korea, the Russian Far East, and Miyagi (northern Japan) being sister (97%) to *P. takesimensis* on Ulleung Island are reasonably well established (Figure 2A), this study raises an issue for the species concept of *P. sargentii* and its close relatives. The ML tree suggests that *P. sargentii* sampled from Jeju Island, South Korea, does not cluster with other conspecific populations, but are embedded within the clade comprising primarily *P. serrulata* (i.e., *P. serrulata* var. *spontanea*, *P. serrulata* var. *pubescens*, and *P. serrulata* var. *quelpaertensis*) and *P. sargentii* var. *verecunda* (Figure 2A). In addition, *P. serrulata* and three infraspecific taxa are shown to be non-monophyletic. Some diagnostic features for delimitation of these infraspecific taxa can be quite variable if not subtle, and thus further detailed morphological study and geographic patterns would be beneficial to revising the species concept of *P. serrulata* and its infraspecific taxa. Although the inflorescence type based on the peduncle length, i.e., umbel or corymb, can be useful for delimiting two closely related species, *P. sargentii* and *P. serrulata* (Chang et al., 2004), this study also suggests that the species concept of *P. sargentii* may require further revision. The non-monophyly of *P*. *sargentii* revealed in this study raises the possibility that the clade A populations distributed in the high mountains of South Korea (excluding Jeju Island), northern Japan, and the Russian Far East, may constitute the species *P. sargentii* as originally described (Rehder, 1908). *Prunus sargentii* populations in Jeju Island, South Korea and southern Japan (e.g., Okayama) may represent *P. serrulata* or the results of introgression between the two closely related taxa. A future morphological and fine-scale molecular phylogenetic study is required to further unravel species delimitation as well as potential gene flow histories of flowering cherries in East Asia.

### Genetic Consequences of Anagenesis in *P. takesimensis*

Theoretically, high levels of genetic variation without geographic partitioning within the island population are predicted as the emerging patterns of the genetic consequences of the species originating from anagenetic speciation as well as a clear genetic distinction between continental progenitor and insular derivative species. This is because the established founding population increases in size over time in a favorable uniform environment on the island, accumulating genetic diversity in the isolated island populations through mutation and recombination, and no geographic partitioning of genetic variation further maintains high genetic levels (Stuessy et al., 2006, 2014; Takayama et al., 2015). Several species pairs in Ulleung Island showed the expected trends with higher (*Dystaenia takesimana* and *D. ibukiensis*) or slightly lower (*Acer takesimense* and *A. pseudosieboldianum*, and *Acer okomotoanum* and *A. mono*) genetic variations in island populations than continental progenitors without geographic partitioning (Pfosser et al., 2002, 2005; Takayama et al., 2012, 2013). The endemic tree species to the Juan Fernández Islands in the Pacific Ocean, *Drimys confertifolia*, *Myrceugenia fernandeziana*, and *Myrceugenia schulzei* also showed the genetic patterns compatible with the hypothesis for anagenesis, i.e., similar level of genetic diversity as their continental progenitors, respectively (*Drimys winteri, Drimys andina*, and *Myrceugenia colchaguensis*) along with no geographical partitioning of the those variations over the Islands (López-Sepúlveda et al., 2013, 2015). Previous studies mostly employed nuclear microsatellites in addition to cpDNA sequences and AFLP analysis to investigate the genetic consequences of anagenesis in *Acer* (nine loci), *Myrceugenia* (six loci), and *Drimys* (nine loci) species. *Prunus* species, specifically belonging to *P. serrulata*/*P. sargentii* complex, appeared more complicated genetically than those species, as they were not clearly distinguished based on 11 nuclear microsatellite loci genotyping (Cho, M.-S., unpublished data). In this study, we assessed the genetic consequences of anagenetic speciation of *P. takesimensis* using different types of molecular data, that is, maternally inherited cpDNA haplotypes and genome-wide SNPs from MIG-seq analysis. No geographic partitioning of genetic variation was found within the populations of *P. takesimensis* in either analysis. The genetic diversity in *P. takesimensis* was lower than the one in *P. sargentii* in haplotype richness, polymorphic sites, gene diversity and nucleotide diversity from cpDNA haplotypes, but was slightly higher in heterozygosity (He) and nucleotide diversity (π) from much more extensive genome-wide SNP analysis (Tables 2, 4). This is consistent with other endemic species derived by anagenesis in Ulleung Island and the Juan Fernández Islands. Generally, oceanic island populations are expected to lose genetic variation at the foundation, as shown in a significant majority of island populations (165 of 202 comparisons) with less allozyme variations at 29% average reduction than their mainland counterparts, as they typically have smaller population sizes than usually larger and broadly distributed mainland progenitor populations (Frankham, 1997). Pfosser et al. (2005) claimed that *D. takesimana* regained genetic diversity by accumulating genetic variations through mutation, recombination, and drift during or after anagenetic speciation along with an increase in population size. In case of *P. takesimensis*, the genome-wide SNPs analyzed by MIG-seq showed a slight increase in genetic diversity, that is, H_*E*_ (o) and H_*E*_ (e): the insular derivative *P. takesimensis* (mean 0.1543 and 0.1905) versus the continental progenitor *P. sargentii* (mean 0.1241 and 0.1658) (Table 4). We, however, cannot rule out the possibility that these results are due to uneven sampling of the two species and further study is required.

The absence of (or very weak) geographic structuring among populations of *P. takesimensis* across Ulleung Island is clearly confirmed by phylogenetic and population genetic structure analyses based on genome-wide SNPs, which is consistent with the expected pattern for anagenetically derived endemic species (Figures 2B, 6). Lack of spatial structure was also corroborated by the AMOVA result of maternally inherited cpDNA haplotypes, which showed much lower genetic variation among populations of *P. takesimensis* than other endemics, 16.30%; 69.52% for *Phedimus takesimensis* (Seo et al., 2020), 51.90% for *Campanula takesimana* (Cheong et al., 2020), and 56.86% for *Rubus takesimensis* (Yang et al., 2019). Gene flow through dispersal of seeds and pollen is a fundamental determinant of spatial genetic structure in natural populations of trees at different spatial scales (Roser et al., 2017). In the absence of spatial genetic structures in *P. takesimensis*, constant gene flow presumably plays a relevant role in reducing genetic differentiation among populations. Flowering cherries show tremendous ability for pollen-mediated gene movement facilitated by their characteristics, such as genetic bridging capacity, inter- and intra-specific genetic compatibility, high frequency of open pollination, perennial nature, tendency to escape from cultivation, and the abundant existence of ornamental and roadside cherries (Cici and Van Acker, 2010). Moreover, it is highly conceivable that genetic exchange by long-distance dispersal of seeds mediated by birds may also occur frequently among the populations of *P. takesimensis* on Ulleung Island.

Other groups of species pairs of progenitor and derivative endemic species on Ulleung Island displayed variable genetic patterns depending on the species, as described previously. Genetic variation on islands is determined by the effects of various factors, such as loss at foundation, subsequent loss caused by finite population size since foundation, and gains arising from secondary immigration and new mutations (Jaenike, 1973; Frankham, 1997). The species pair of an endemic shrub, *Rubus takesimensis*, and its continental progenitor, *R. crataegifolius*, showed significantly higher genetic diversity statistics in the continental progenitor *R. crataegifolius* in a few parameters (e.g., number of haplotypes and nucleotide diversity) than the insular derivative *R. takesimensis*, even though *R. takesimensis* does not have low levels of genetic variation because it has experienced multiple introductions from geographically and genetically diverse source populations (Yang et al., 2019). Two herbaceous endemic plants of *Campanula takesimana* and *Phedimus takesimensis* contrasted with each other in genetic diversity statistics; there was no apparent genetic diversity reduction in *Phedimus takesimensis* compared to its continental progenitor, *P. kamtchaticus*; however, *Campanula takesimana* had substantially lower genetic diversity than *C. punctata* in the South Korean Peninsula. In terms of the partitioning of the genetic variations on the Island, both endemic plants were clearly structured, unlike other herbaceous or woody endemic plants anagenetically derived on Ulleung Island. *Campanula takesimana* showed substantial genetic structuring and a very narrow geographical source area, that is, Bonghwa in the Korean Peninsula via the plausible stepping stone of Dokdo Island (Cheong et al., 2020). The seed splash mechanism is responsible for the population genetic structure and differentiation among populations of *Phedimus takesimensis* due to the limited seed-mediated gene flow by raindrops with relatively short dispersal distances (Seo et al., 2020). All these compilations of the genetic consequences of anagenetically derived endemic species on Ulleung Island are based on a limited number of studies conducted to date. Given the important role of anagenesis in the origin of Ulleung Island endemic plants, it is critical to investigate a much broader group of progenitor and derivative relationships to gain insights into speciation on oceanic islands.

## Conclusion

We provide the most extensive genetic data to date, revealing the monophyly and some clues to the geographical origin of the wild flowering cherry endemic to Ulleung Island, *P. takesimensis*, from SNPs detected by MIG-seq and complementary cpDNA haplotype analyses. Based on the continental progenitor and insular derivative relationship, *P. takesimensis* endemic to Ulleung Island appears to have been derived anagenetically from some uncertain source population of *P. sargentii* in the Korean Peninsula, Russia and Miyagi in Japan via likely single introduction, although the possibility of multiple introductions cannot be completely ruled out. The genetic differentiation of *P. takesimensis* from its continental progenitor *P. sargentii* is corroborated by comparative analyses of population genetic structure and phylogeny based on MIG-seq data. The emerging patterns of the genetic consequences of anagenesis in *P. takesimensis* correspond to theoretical predictions, as shown in other woody endemics. Generally, higher levels of genetic diversity are found in populations of the continental progenitor *P. sargentii* compared to island populations of *P. takesimensis*. The absence of strong population genetic structuring or geographical patterns in *P. takesimensis*, which is consistent with our expectations, is possibly caused by gene flow assisted by seed dispersal mediated by birds across Ulleung Island. This study also highlights the effectiveness of the MIG-seq method for species delimitation and unraveling the complex evolutionary history of island endemic plant groups. However, the other taxa within the *P. serrulata/P. sargentii* complex remain as unresolved by MIG-seq method, which is not sufficient to distinguish the genetic lineages of sympatric wild flowering cherry species, presumably exchanging genetic materials constantly through hybridization. Considering that MIG-seq utilizes only ISSR regions and provides fewer numbers of SNPs than ddRAD-seq (e.g., ∼1,000 *vs*. ∼100,000 SNPs) (Peterson et al., 2012; Suyama and Matsuki, 2015), future studies require more investigation to overcome the potential limitation of MIG-seq method and clarify the complex evolutionary history of wild flowering cherries.

## Data Availability Statement

The datasets presented in this study can be found in online repositories. The names of the repository/repositories and accession number(s) can be found below: Dryad (doi: 10.5061/dryad.x3ffbg7jn). Raw reads are available in the Short Read Archive (SRA) at NCBI under the bioProject number of PRJNA742508 titled as “262 Prunus MIG-Seq”.

## Author Contributions

M-SC and S-CK designed the experiment. M-SC and MM collected the samples. M-SC, KT, and JY performed the experiments and analyzed the data. M-SC drafted the manuscript. S-CK revised the manuscript. M-SC, KT, JY, MM, and S-CK approved the final manuscript. All authors contributed to the article and approved the submitted version.

## Conflict of Interest

The authors declare that the research was conducted in the absence of any commercial or financial relationships that could be construed as a potential conflict of interest.

## Publisher’s Note

All claims expressed in this article are solely those of the authors and do not necessarily represent those of their affiliated organizations, or those of the publisher, the editors and the reviewers. Any product that may be evaluated in this article, or claim that may be made by its manufacturer, is not guaranteed or endorsed by the publisher.

## Footnotes

1
http://hannonlab.cshl.edu/fastx_toolkit/
