# Supplementary material for: Genome-Wide Single Nucleotide Polymorphism Analysis Elucidates the Evolution of Prunus takesimensis in Ulleung Island: The Genetic Consequences of Anagenetic Speciation
Source: Front Plant Sci. 2021 Sep 2;12:706195. doi: 10.3389/fpls.2021.706195 (PMC8445234; doi:10.3389/fpls.2021.706195)
Supplement: Supplementary file 1 [file Data_Sheet_1.docx]

Supplementary Material

## Supplementary Figures


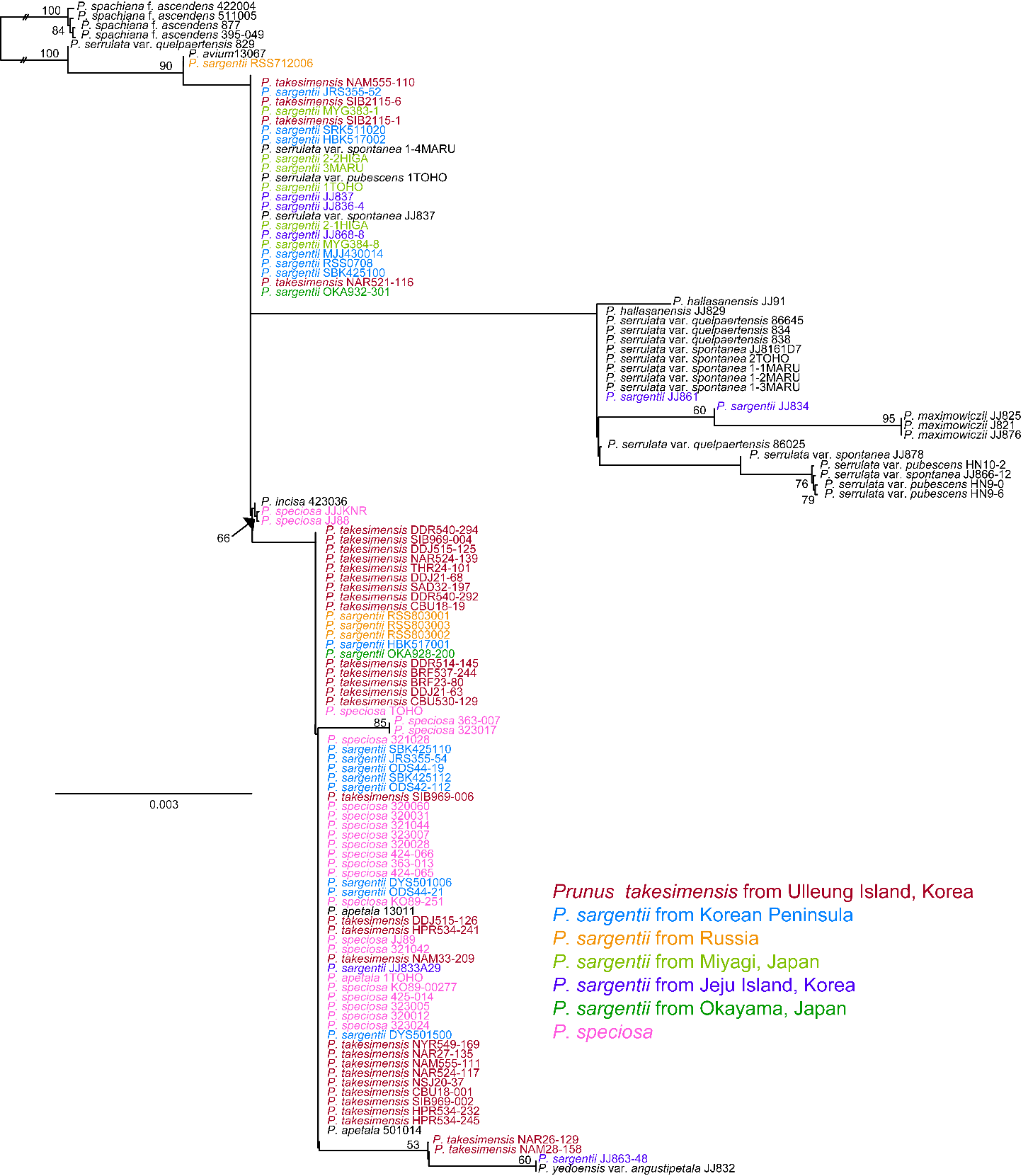
**Figure S1.** Maximum likelihood tree produced by IQ-TREE based on concatenated nrDNA ITS and ETS regions, including 123 accessions of flowering cherries of the subgenus *Cerasus* (the genus *Prunus*). Color codes for species and geographical regions: red for *P. takesimensis* from Ulleung Island, blue for *P. sargentii* from Korean Peninsula, orange for *P. sargentii* from Russia, light green for *P. sargentii* from Miyagi, Japan, purple for *P. sargentii* from Jeju Island, Korea, dark green for *P. sargentii* from Okayama, Japan, and pink for *P. spesiosa*. Numbers above branches indicate bootstrap support (BS) percentages of >50%.

**
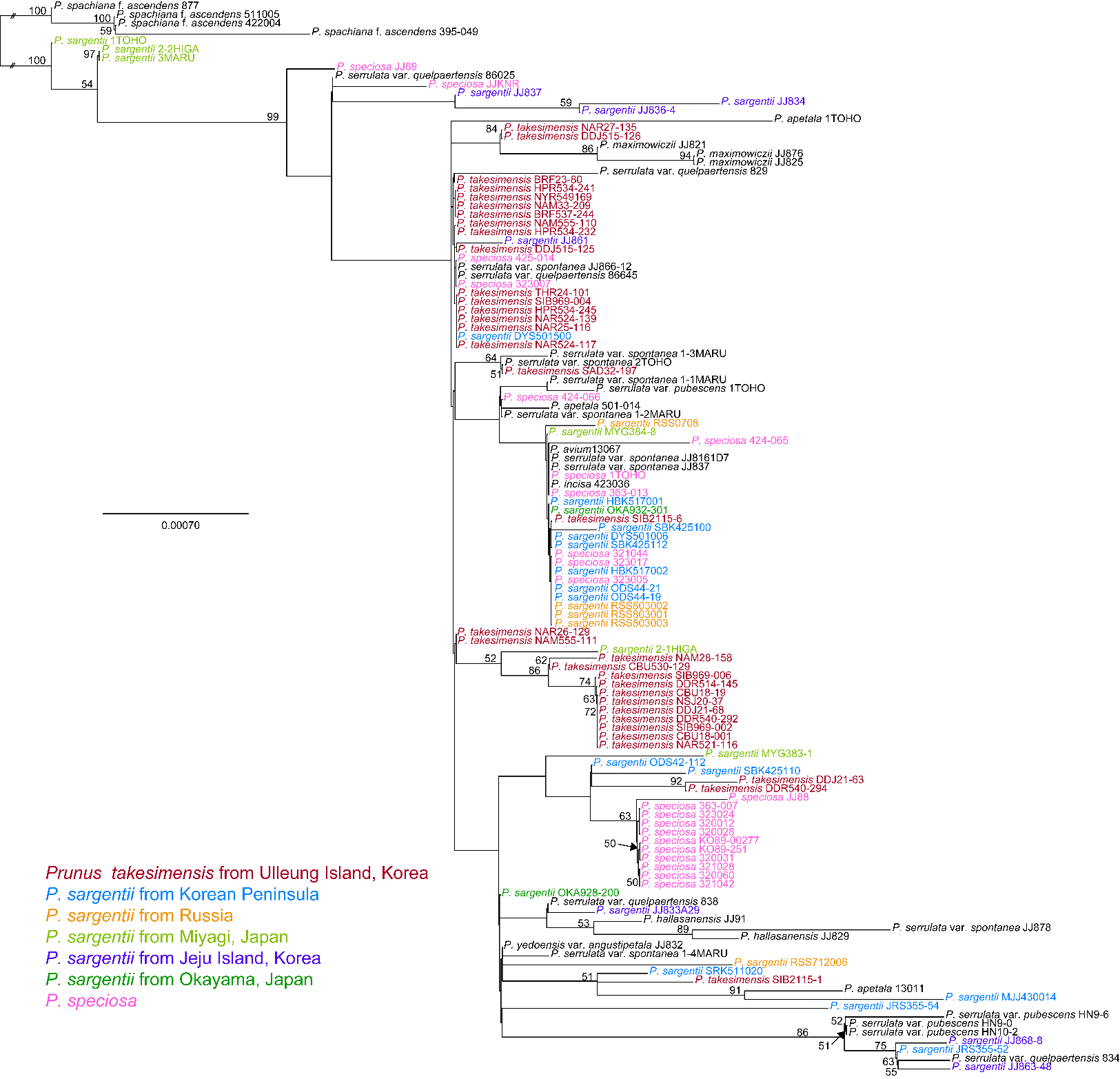
**

**Figure S2**. Maximum likelihood tree produced by IQ-TREE based on concatenated seven cpDNA regions, including 123 accessions of flowering cherries of the subgenus *Cerasus* (the genus *Prunus*). Color codes for species and geographical regions: red for *P.* *takesimensis* from Ulleung Island, blue for *P.* *sargentii* from Korean Peninsula, orange for *P.* *sargentii* from Russia, light green for *P.* *sargentii* from Miyagi, Japan, purple for *P.* *sargentii* from Jeju Island, Korea, dark green for *P.* *sargentii* from Okayama, Japan, and pink for *P. spesiosa*. Numbers above branches indicate bootstrap support (BS) percentages of >50%.


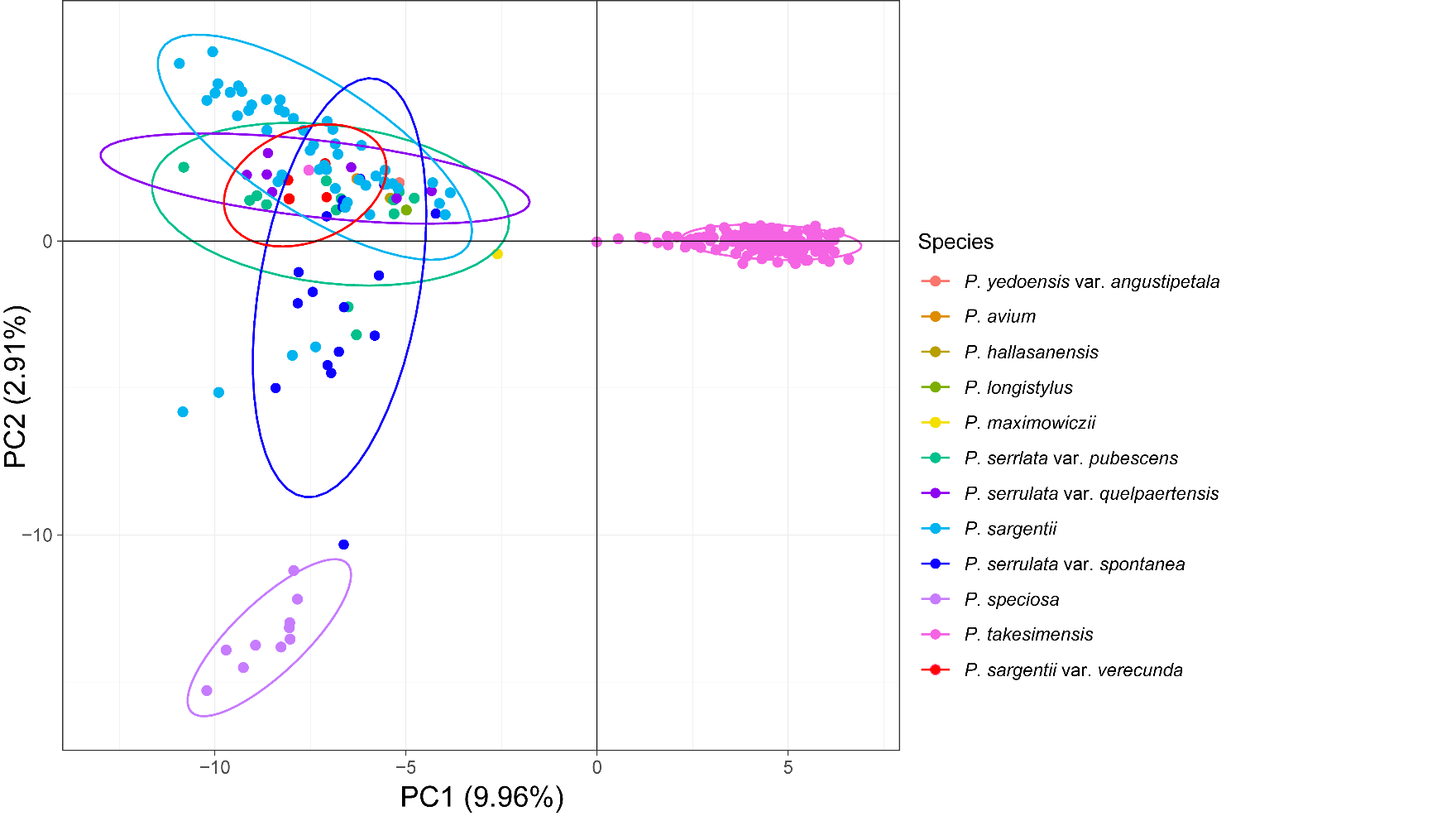


**Figure S3**. Score plot for principal component analysis of 262 *Cerasus* flowering cherries performed using R based on the SNPs detected by MIG-seq analysis. Different colors denote different species as specified on the plot.

## Supplementary Table

**Table S1.** List of plant materials used for multiple phylogenetic analyses in this study. Summary per species and detailed list of total materials are specified.

**A. Summary per species**

| **Species** | **Population** | **Locality** | **nrDNA/cpDNA phylogeny** | **SNP (MIG-seq) analysis** | **cpDNA network analysis** |
| --- | --- | --- | --- | --- | --- |
|  |  |  |  |  |  |
| ***Prunus takesimensis* Nakai from Ulleung Island, Korea** | CBU | Cheonbu | 3 | 10 | 5 |
|  | CHU | Chusan |  | 14 | 5 |
|  | DDJ | Dokdo Jeonmangdae | 4 | 12 | 5 |
|  | HGM | Hyangmok Jeonmangdae |  | 14 | 5 |
|  | HPR | Hyunpo-ri | 3 | 12 | 5 |
|  | JRG | Joongryong |  | 14 | 5 |
|  | MAL | Maljandeung |  | 10 | 5 |
|  | NAM | Namseo-ri | 4 | 9 | 5 |
|  | NAR | Nari | 6 | 11 | 6 |
|  | NRH* | Nari, with hairs |  | 10 |  |
|  | NSJ | Naesujeon | 1 | 5 | 5 |
|  | SAD | Sa-dong | 1 | 9 | 5 |
|  | SIB | Seonginbong | 5 | 19 | 8 |
|  | THR | Taeha-ri | 1 | 10 | 5 |
|  |  | others | 6 | 3 |  |
|  |  | **subtotal** | **34** | **162** | **69** |
| ***Prunus sargentii* Rehder** | JJ | Jeju Island, Korea | 7 | 10 | 7 |
|  | ODS | Mt. Odaesan, Korean Peninsula | 3 | 11 | 5 |
|  | RSS | Russia | 5 | 4 | 5 |
|  | MYG | Miyagi, Japan | 6 | 12 | 8 |
|  | OKA | Okayama, Japan | 2 | 4 | 5 |
|  |  | Other mountains in Korean Peninsula | 11 | 5 |  |
|  |  | **subtotal** | **34** | **46** | **30** |
| ***Prunus maximowiczii* Ruprecht** |  | Jeju Island, Korea | 3 | 1 |  |
| ***Prunus avium* (L.) L.** |  | Aomori, Japan | 1 | 1 |  |
| ***Prunus spachiana* f. *ascendens* (Makino) Kitam.** |  | Jeju Island, Korea | 3 |  |  |
|  |  | Miyagi, Japan | 1 |  |  |
| ***Prunus incisa* Thunb.** |  | Miyagi, Japan | 1 |  |  |
| ***Prunus apetala* (Siebold & Zucc.) Franch & Sav.** |  | Miyagi, Japan | 3 |  |  |
| ***Prunus serrulata* var. *spontanea* (Maxim.) E.H.Wilson** |  | Jeju Island, Korea | 4 | 5 |  |
|  |  | Haenam, Korean Peninsula |  | 2 |  |
|  |  | Other islands, Korea |  | 8 |  |
|  |  | Miyagi, Japan | 5 |  |  |
| ***Prunus serrlata* var. *pubescens* (Makino)** |  | Mountains in Korean Peninsula | 3 | 11 |  |
|  |  | Miyagi, Japan | 1 |  |  |
|  |  | Korean islands |  | 2 |  |
| ***Prunus serrulata* var. *quelpaertensis* (Nakai) Uyeki** |  | Jeju Island, Korea | 5 | 7 |  |
| ***Prunus yedoensis* M. var. *angustipetala* Kim et Kim** |  | Jeju Island, Korea | 1 | 1 |  |
| ***Prunus hallasanensis* Kim et Kim** |  | Jeju Island, Korea | 2 | 1 |  |
| ***Prunus longistylus* Kim et Kim** |  | Jeju Island, Korea |  | 1 |  |
| ***Prunus speciosa* (Koidz.) Ingram** |  | Jeju Island, Korea | 3 |  |  |
|  |  | Tokyo and Miyagi, Japan | 8 | 1 |  |
|  |  | Miyagi, Japan | 4 |  |  |
|  |  | Izu, Japan | 11 | 9 |  |
| ***Prunus sargenti*i var. *verecunda* (Koidz.) Chin S. Chang** |  | Mountains in Korean Peninsula |  | 4 |  |
| **Other species** |  | **subtotal** | **55** | **54** |  |
|  |  |  |  |  |  |
| **Total** |  |  | **123** | **262** | **99** |

**B. Details of plant materials used for three analyses performed in this study.**

| **Species/ Accession No.** | **Collection site** | **Collection Date** | **Specimen No.** | **123 *Prunus* ITS/ETS, 7 cpDNA Phylogeny** | **281 *Prunus* MigSeq analysis** | **99 *Prunus* 5 cpDNA Network analysis** |  |  |  |  |  |  |  |  |
| --- | --- | --- | --- | --- | --- | --- | --- | --- | --- | --- | --- | --- | --- | --- |
| **Subgenus *Cerasus*, Genus *Prunus*, Rosaceae** | |  |  | | | |  |  | |  | | | |  |
| ***Prunus maximowiczii* Ruprecht, section *Phyllomahaleb* Koehne** | |  |  | | | |  |  | |  | | | |  |
| MX_JJ876 | Bangseongyo, Jeju Island, Korea | 03-May-11 | *SKK Cho et al. 110503163* | o |  |  |  |  |  |  |  |  |  |  |
| MX_JJ82-5 | Witseoreum, Jeju Island, Korea | 09-May-12 | *SKK Cho et al. 120509165* | o |  |  |  |  |  |  |  |  |  |  |
| MX_JJ82-1 | Witseoreum, Jeju Island, Korea | 09-May-12 | *SKK Cho et al. 120509164* | o | o |  |  |  |  |  |  |  |  |  |
|  |  |  |  |  |  |  |  |  |  |  |  |  |  |  |
| ***Prunus avium* (L.) L., section *Eucerasus* Koehne** | |  |  | | | |  |  | |  | | | |  |
| AV_13067 | Aomori, Japan | 2013-05-10 | *SKK Cho et al. 13067* | o | o |  |  |  |  |  |  |  |  |  |
|  |  |  |  |  |  |  |  |  |  |  |  |  |  |  |
| **Section *Pseudocerasus* Koehne*Prunus spachiana* f. *ascendens* (Makino) Kitam.** | | |  | | | |  | |  | |  | | | |
| PE_422004 | Bonggae Wangbeot Natural Stand, Jeju Island, Korea | 22-Apr-12 | *SKK Cho et al. 120422004* | o |  |  |  |  |  |  |  |  |  |  |
| PE_511005 | Youngpyung-dong, Jeju Island, Korea | 11-May-12 | *SKK Cho et al. 120511005* | o |  |  |  |  |  |  |  |  |  |  |
| PE_877 | Eorimok, Jeju Island, Korea | 03-May-11 | *SKK Cho et al. 110503135* | o |  |  |  |  |  |  |  |  |  |  |
| PP_395-049 | Miyagi, Japan | 24-Apr-13 | *SKK Cho et al. 130424202* | o |  |  |  |  |  |  |  |  |  |  |
|  |  |  |  |  |  |  |  |  |  |  |  |  |  |  |
| ***Prunus incisa* Thunb., section *Pseudocerasus* Koehne** | |  |  | | | |  |  | |  | | | |  |
| IN_423036 | Tohoku Univ. Botanical Garden, Sendai, Japan | 23-Apr-13 | *SKK Cho et al. 130423036* | o |  |  |  |  |  |  |  |  |  |  |
|  |  |  |  |  |  |  |  |  |  |  |  |  |  |  |
| ***Prunus apetala (*Siebold & Zucc.) Franch & Sav., section *Pseudocerasus* Koehne** | | |  | | | |  | |  | |  | | | |
| PA_13011 | Tochigi, Japan | 02-May-13 | *SKK Cho et al. 13011* | o |  |  |  |  |  |  |  |  |  |  |
| PA_1TOHO | Tohoku Univ. Botanical Garden, Sendai, Japan | 08-May-12 | None | o |  |  |  |  |  |  |  |  |  |  |
| PA_501014 | Tsuruoka, Japan | 01-May-13 | *SKK Cho et al. 130501014* | o |  |  |  |  |  |  |  |  |  |  |
|  |  |  |  |  |  |  |  |  |  |  |  |  |  |  |
| ***Prunus serrulata* var. *spontanea* (Maxim.) E.H.Wilson, section *Pseudocerasus* Koehne** | | | | | | |  | |  | | |  |  |  |
| SE_JJ8161D7 | Hannam-ri, Jeju Island, Korea | 13-Apr-11 | *SKK Cho et al. 110413148* | o |  |  |  |  |  |  |  |  |  |  |
| SE_JJ837 | Sancheondan, Jeju Island, Korea | 19-Apr-11 | *SKK Cho et al. 110419149* | o | o |  |  |  |  |  |  |  |  |  |
| SE_JJ866-12 | Seogwipo Natural Recreation Forest, Jeju Island, Korea | 02-May-11 | *SKK Cho et al. 110502150* | o | o |  |  |  |  |  |  |  |  |  |
| SE_JJ878 | Young-dong, Jeju Island, Korea | 03-May-11 | *SKK Cho et al. 110503151* | o | o |  |  |  |  |  |  |  |  |  |
| SE_JJ802 | Dosun-dong, Jeju Island, Korea | 12-Apr-11 | *SKK Cho et al. 110412147* |  | o |  |  |  |  |  |  |  |  |  |
| SE_JJ816 | Hannam-ri, Jeju Island, Korea | 13-Apr-11 | *SKK Cho et al. 110413816* |  | o |  |  |  |  |  |  |  |  |  |
| SE_HN41771 | Haenam, Korea | 17-Apr-12 | *SKK Cho et al. 120417071* |  | o |  |  |  |  |  |  |  |  |  |
| SE_HN4178 | Haenam, Korea | 17-Apr-12 | *SKK Cho et al. 120417008* |  | o |  |  |  |  |  |  |  |  |  |
| SE_WD41813 | Wan Island, Korea | 18-Apr-12 | *SKK Cho et al. 120418013* |  | o |  |  |  |  |  |  |  |  |  |
| SE_WD412051 | Wan Island, Korea | 12-Apr-12 | *SKK Cho et al. 120412051* |  | o |  |  |  |  |  |  |  |  |  |
| SE_BGD41762 | Bogil Island, Korea | 17-Apr-12 | *SKK Cho et al. 120417062* |  | o |  |  |  |  |  |  |  |  |  |
| SE_BGD41766 | Bogil Island, Korea | 17-Apr-12 | *SKK Cho et al. 120417066* |  | o |  |  |  |  |  |  |  |  |  |
| SE_GJD4215 | Geoje Island, Korea | 21-Apr-12 | *SKK Cho et al. 120421005* |  | o |  |  |  |  |  |  |  |  |  |
| SE_GJD42122 | Geoje Island, Korea | 21-Apr-12 | *SKK Cho et al. 120421022* |  | o |  |  |  |  |  |  |  |  |  |
| SE_GMD4241 | Geomun Island, Korea | 24-Apr-16 | *SKK Cho et al. 160424001* |  | o |  |  |  |  |  |  |  |  |  |
| SE_GMD4245 | Geomun Island, Korea | 24-Apr-16 | *SKK Cho et al. 160424005* |  | o |  |  |  |  |  |  |  |  |  |
| SE_GMD42491 | Geomun Island, Korea | 24-Apr-16 | *SKK Cho et al. 160424091* |  | o |  |  |  |  |  |  |  |  |  |
| SE_1-1MARU | Miyagi, Japan | 29-Apr-12 | None | o |  |  |  |  |  |  |  |  |  |  |
| SE_1-2MARU | Miyagi, Japan | 29-Apr-12 | None | o |  |  |  |  |  |  |  |  |  |  |
| SE_1-3MARU | Miyagi, Japan | 29-Apr-12 | None | o |  |  |  |  |  |  |  |  |  |  |
| SE_1-4MARU | Miyagi, Japan | 29-Apr-12 | None | o |  |  |  |  |  |  |  |  |  |  |
| SE_2TOHO | Miyagi, Japan | 29-Apr-12 | None | o |  |  |  |  |  |  |  |  |  |  |
|  |  |  |  |  |  |  |  |  |  |  |  |  |  |  |
| ***Prunus serrlata* var. *pubescens* (Makino), section *Pseudocerasus* Koehne** | | |  | | | |  | |  | |  | | | |
| PB_HN9-0 | Dooryunsan Wang Beot Natural Stand, Haenam, Korea | 17-Apr-12 | *SKK Cho et al. 120417136* | o |  |  |  |  |  |  |  |  |  |  |
| PB_HN9-6 | Dooryunsan Wang Beot Natural Stand, Haenam, Korea | 17-Apr-12 | *SKK Cho et al. 120417153* | o |  |  |  |  |  |  |  |  |  |  |
| PB_HN10-2 | Dooryunsan Wang Beot Natural Stand, Haenam, Korea | 17-Apr-12 | *SKK Cho et al. 120417137* | o |  |  |  |  |  |  |  |  |  |  |
| PB_1TOHO | Tohoku Univ. Botanical Garden, Sendai, Japan | 08-May-12 | None | o |  |  |  |  |  |  |  |  |  |  |
| PB_WD4189 | Wan Island, Korea | 18-Apr-12 | *SKK Lim et al. 120418009* |  | o |  |  |  |  |  |  |  |  |  |
| PB_JSS4318 | Jeoksangsan, Korea | 30-Apr-12 | *SKK Lim et al. 120430018* |  | o |  |  |  |  |  |  |  |  |  |
| PB_DDS5011 | Daedunsan, Korea | 01-May-12 | *SKK Lim et al. 120501001* |  | o |  |  |  |  |  |  |  |  |  |
| PB_THS50414 | Taehwasan, Korea | 04-May-12 | *SKK Lim et al. 120504014* |  | o |  |  |  |  |  |  |  |  |  |
| PB_TBK5053 | Taebaiksan, Korea | 05-May-12 | *SKK Lim et al. 120505003* |  | o |  |  |  |  |  |  |  |  |  |
| PB_DTS50528 | Dutasan, Korea | 12-May-12 | *SKK Lim et al. 120505028* |  | o |  |  |  |  |  |  |  |  |  |
| PB_DGR51240 | Daekwanryong, Korea | 05-May-12 | *SKK Lim et al. 120512040* |  | o |  |  |  |  |  |  |  |  |  |
| PB_NJS4132 | Naejangsan, Korea | 13-Apr-13 | *SKK Cho et al. 130413002* |  | o |  |  |  |  |  |  |  |  |  |
| PB_NJS4144 | Naejangsan, Korea | 14-Apr-13 | *SKK Cho et al. 130414004* |  | o |  |  |  |  |  |  |  |  |  |
| PB_GGS50650 | Gwanggyosan, Korea | 06-May-13 | *SKK Cho et al. 130506050* |  | o |  |  |  |  |  |  |  |  |  |
| PB_GDS4223 | Geomdansan, Korea | 22-Apr-15 | *SKK Cho et al. 150422300* |  | o |  |  |  |  |  |  |  |  |  |
| PB_SBK42518 | Sobaeksan, Korea | 25-Apr-15 | *SKK Cho et al. 150425108* |  | o |  |  |  |  |  |  |  |  |  |
| PB_JD16041 | Jin Island, Korea | 01-Apr-16 | *SKK JJH et al. 160401046* |  | o |  |  |  |  |  |  |  |  |  |
|  |  |  |  |  |  |  |  |  |  |  |  |  |  |  |
| ***Prunus serrulata* var. *quelpaertensis* (Nakai) Uyeki*,* section *Pseudocerasus* Koehne** | | |  | | | |  | |  | |  | | | |
| QP_829 | Hannam Experimental Forest, Jeju Island, Korea | 18-Apr-11 | *SKK Cho et al. 110418154* | o |  |  |  |  |  |  |  |  |  |  |
| QP_834 | 1^st^ Sallok Rd., Jeju Island, Korea | 19-Apr-11 | *SKK Cho et al. 110419155* | o |  |  |  |  |  |  |  |  |  |  |
| QP_838 | Sancheondan, Jeju Island, Korea | 19-Apr-11 | *SKK Cho et al. 110419156* | o |  |  |  |  |  |  |  |  |  |  |
| QP_86025 | 1100 Resting Area, Jeju Island, Korea | 02-May-11 | *SKK Cho et al. 110502157* | o | o |  |  |  |  |  |  |  |  |  |
| QP_86645 | Seogwipo Natural Recreational Forest, Jeju Island, Korea | 02-May-11 | *SKK Cho et al. 110502158* | o | o |  |  |  |  |  |  |  |  |  |
| QP_23012 | Seongpangyo, Jeju Island, Korea | 23-Apr-12 | *SKK Cho et al. 120423012* |  | o |  |  |  |  |  |  |  |  |  |
| QP_32011 | Hanbalgyo, Jeju Island, Korea | 24-Apr-12 | *SKK Cho et al. 120424011* |  | o |  |  |  |  |  |  |  |  |  |
| QP_8602 | 1100 Resting Area, Jeju Island, Korea | 02-May-11 | *SKK Cho et al. 11052602* |  | o |  |  |  |  |  |  |  |  |  |
| QP_8661 | Seogwipo Natural Recreational Forest, Jeju Island, Korea | 02-May-11 | *SKK Cho et al. 11052601* |  | o |  |  |  |  |  |  |  |  |  |
| QP_96-009 | Youngyyeong-dong, Jeju Island, Korea | 11-May-12 | *SKK Cho et al. 120511009* |  | o |  |  |  |  |  |  |  |  |  |
|  |  |  |  |  |  |  |  |  |  |  |  |  |  |  |
| ***Prunus yedoensis* M. var. *angustipetala* Kim et Kim, section *Pseudocerasus* Koehne** | | |  | | | |  | |  | |  | | | |
| AP_JJ832 | Kwaneumsa, Jeju Island, Korea | 19-Apr-11 | *SKK Cho et al. 110419159* | o | o |  |  |  |  |  |  |  |  |  |
|  |  |  |  |  |  |  |  |  |  |  |  |  |  |  |
| ***Prunus hallasanensis* Kim et Kim, section *Pseudocerasus* Koehne** | |  |  | | | |  |  | |  | | | |  |
| HL_JJ91 | Ara Sameuiak Trekking course, Jeju Island, Korea | 10-May-12 | *SKK Cho et al. 120510161* | o |  |  |  |  |  |  |  |  |  |  |
| HL_JJ829 | Hannam Experimental Forest, Jeju Island, Korea | 18-Apr-11 | *SKK Cho et al. 110418160* | o | o |  |  |  |  |  |  |  |  |  |
|  |  |  |  |  |  |  |  |  |  |  |  |  |  |  |
| ***Prunus longistylus* Kim *et* Kim collected from Jeju Island, Korea, section *Pseudocerasus* Koehne** | | | | | | |  | |  | | |  |  |  |
| LS_JJ874 | Tamra Valley, Jeju Island, Korea | 03-May-11 | *SKK Cho et al. 110503162* |  | o |  |  |  |  |  |  |  |  |  |
|  |  |  |  |  |  |  |  |  |  |  |  |  |  |  |
| ***Prunus speciosa* (Koidz.) Ingram, section *Pseudocerasus* Koehne** | |  |  | | | |  |  | |  | | | |  |
| SP_JJKNR | Gimnyeong-ri, Jeju Island, Korea | 25-Oct-11 | *SKK Cho et al. 111025166* | o |  |  |  |  |  |  |  |  |  |  |
| SP_JJ88 | Gimnyeong-ri, Jeju Island, Korea | 10-May-12 | *SKK Cho et al. 120510167* | o |  |  |  |  |  |  |  |  |  |  |
| SP_JJ89 | Gimnyeong-ri, Jeju Island, Korea | 10-May-12 | *SKK Cho et al. 120510168* | o |  |  |  |  |  |  |  |  |  |  |
| SP_KO89-251 | Koishikawa Botanical Garden, Tokyo, Japan | 18-Apr-13 | None | o | o |  |  |  |  |  |  |  |  |  |
| SP_KO89-00277 | Koishikawa Botanical Garden, Tokyo, Japan | 18-Apr-13 | None | o |  |  |  |  |  |  |  |  |  |  |
| SP_1TOHO | Tohoku Univ. Botanical Garden, Sendai, Japan | 20-Apr-12 | None | o |  |  |  |  |  |  |  |  |  |  |
| SP_363-007 | Hakone, Japan | 20-Apr-13 | *SKK Cho et al. 130420007* | o |  |  |  |  |  |  |  |  |  |  |
| SP_363-013 | Hakone, Japan | 20-Apr-13 | *SKK Cho et al. 130420013* | o |  |  |  |  |  |  |  |  |  |  |
| SP_425-014 | Tohoku Univ. Botanical Garden, Sendai, Japan | 25-Apr-13 | *SKK Cho et al. 130425014* | o |  |  |  |  |  |  |  |  |  |  |
| SP_424-065 | Tohoku Univ. Botanical Garden, Sendai, Japan | 24-Apr-13 | *SKK Cho et al. 130424065* | o |  |  |  |  |  |  |  |  |  |  |
| SP_424-066 | Tohoku Univ. Botanical Garden, Sendai, Japan | 24-Apr-13 | *SKK Cho et al. 130424066* | o |  |  |  |  |  |  |  |  |  |  |
| SP_320012 | Oshima Island, Japan | 20-Mar-15 | *SKK Cho et al. 150320012* | o |  |  |  |  |  |  |  |  |  |  |
| SP_320028 | Oshima Island, Japan | 20-Mar-15 | *SKK Cho et al. 150320028* | o |  |  |  |  |  |  |  |  |  |  |
| SP_320031 | Oshima Island, Japan | 20-Mar-15 | *SKK Cho et al. 150320031* | o |  |  |  |  |  |  |  |  |  |  |
| SP_320060 | Oshima Island, Japan | 20-Mar-15 | *SKK Cho et al. 150320060* | o |  |  |  |  |  |  |  |  |  |  |
| SP_321028 | Kozushima Island, Japan | 21-Mar-15 | *SKK Cho et al. 150321028* | o |  |  |  |  |  |  |  |  |  |  |
| SP_321042 | Kozushima Island, Japan | 21-Mar-15 | *SKK Cho et al. 150321042* | o |  |  |  |  |  |  |  |  |  |  |
| SP_321044 | Kozushima Island, Japan | 21-Mar-15 | *SKK Cho et al. 150321044* | o | o |  |  |  |  |  |  |  |  |  |
| SP_323005 | Izu Peninsula, Japan | 23-Mar-15 | *SKK Cho et al. 150323005* | o | o |  |  |  |  |  |  |  |  |  |
| SP_323007 | Izu Peninsula, Japan | 23-Mar-15 | *SKK Cho et al. 150323007* | o |  |  |  |  |  |  |  |  |  |  |
| SP_323017 | Izu Peninsula, Japan | 23-Mar-15 | *SKK Cho et al. 150323017* | o |  |  |  |  |  |  |  |  |  |  |
| SP_323024 | Izu Peninsula, Japan | 23-Mar-15 | *SKK Cho et al. 150323024* | o | o |  |  |  |  |  |  |  |  |  |
| SP_320019 | Oshima Island, Japan | 20-Mar-15 | *SKK Cho et al. 150320019* |  | o |  |  |  |  |  |  |  |  |  |
| SP_320047 | Oshima Island, Japan | 20-Mar-15 | *SKK Cho et al. 150320047* |  | o |  |  |  |  |  |  |  |  |  |
| SP_321002 | Kozushima Island, Japan | 21-Mar-15 | *SKK Cho et al. 150321002* |  | o |  |  |  |  |  |  |  |  |  |
| SP_321038 | Kozushima Island, Japan | 21-Mar-15 | *SKK Cho et al. 150321038* |  | o |  |  |  |  |  |  |  |  |  |
| SP_321062 | Kozushima Island, Japan | 21-Mar-15 | *SKK Cho et al. 150321062* |  | o |  |  |  |  |  |  |  |  |  |
| SP_323003 | Izu Peninsula, Japan | 23-Mar-15 | *SKK Cho et al. 150323003* |  | o |  |  |  |  |  |  |  |  |  |
|  |  |  |  |  |  |  |  |  |  |  |  |  |  |  |
| ***Prunus sargentii* Rehder, section *Pseudocerasus* Koehne** | |  |  | | | |  |  | |  | | | |  |
| SA_JJ2110 | Seongpangyo, Jeju Island, Korea | 23-Apr-12 | *SKK Cho et al. 120423010* |  | o |  |  |  |  |  |  |  |  |  |
| SA_JJ831 | Dosundong, Jeju Island, Korea | 18-Apr-11 | *SKK Cho et al. 110418305* |  | o |  |  |  |  |  |  |  |  |  |
| SA_JJ832 | Kwaneumsa, Jeju Island, Korea | 19-Apr-11 | *SKK Cho et al. 110419832* |  | o |  |  |  |  |  |  |  |  |  |
| SA_JJ8338 | Kwaneumsa, Jeju Island, Korea | 19-Apr-11 | *SKK Cho et al. 110419833* |  | o |  |  |  |  |  |  |  |  |  |
| SA_JJ833A29 | Kwaneumsa, Jeju Island, Korea | 19-Apr-11 | *SKK Cho et al. 110419138* | o |  | o |  |  |  |  |  |  |  |  |
| SA_JJ834 | 1^st^ Sallok Rd., Jeju Island, Korea | 19-Apr-11 | *SKK Cho et al. 110419139* | o |  | o |  |  |  |  |  |  |  |  |
| SA_JJ836-4 | 1^st^ Sallok Rd., Jeju Island, Korea | 19-Apr-11 | *SKK Cho et al. 110419140* | o | o | o |  |  |  |  |  |  |  |  |
| SA_JJ837 | Sancheondan, Jeju Island, Korea | 19-Apr-11 | *SKK Cho et al. 110419141* | o |  | o |  |  |  |  |  |  |  |  |
| SA_JJ838-4 | Mysterious Road, Jeju Island, Korea | 19_Apr-11 | *SKK Cho et al. 110419203* |  | o |  |  |  |  |  |  |  |  |  |
| SA_JJ857-30 | Youngsil, Jeju Island, Korea | 02-May-11 | *SKK Cho et al. 110502142* |  | o |  |  |  |  |  |  |  |  |  |
| SA_JJ861 | 1100 Rest area, Jeju Island, Korea | 02-May-11 | *SKK Cho et al. 110502201* | o |  | o |  |  |  |  |  |  |  |  |
| SA_JJ862-10 | 1100 Rest area, Jeju Island, Korea | 02-May-11 | *SKK Cho et al. 110502202* |  | o |  |  |  |  |  |  |  |  |  |
| SA_JJ863-48 | Eorimok, Jeju Island, Korea | 02-May-11 | *SKK Cho et al. 110502143* | o |  | o |  |  |  |  |  |  |  |  |
| SA_JJ866 | Seogwipo Natural Recreation Forest, Jeju Island, Korea | 02-May-11 | *SKK Cho et al. 110502613* |  | o |  |  |  |  |  |  |  |  |  |
| SA_JJ868-8 | Sinrye-ri, Wangbeot Natural Stand, Jeju Island, Korea | 03-May-11 | *SKK Cho et al. 110503144* | o | o | o |  |  |  |  |  |  |  |  |
| SA_SRK511020 | Seoraksan, Korea | 11-May-12 | *SKK Cho et al. 120511020* | o |  |  |  |  |  |  |  |  |  |  |
| SA_HBK517001 | Hambaiksan, Korea | 17-May-13 | *SKK Cho et al. 130517001* | o | o |  |  |  |  |  |  |  |  |  |
| SA_HBK517002 | Hambaiksan, Korea | 17-May-13 | *SKK Cho et al. 130517002* | o |  |  |  |  |  |  |  |  |  |  |
| SA_MJJ430014 | Minjujisan, Korea | 30-Apr-14 | *SKK Cho et al. 140430014* | o |  |  |  |  |  |  |  |  |  |  |
| SA_DYS501006 | Deogyusan, Korea | 01-May-14 | *SKK Cho et al. 140501006* | o |  |  |  |  |  |  |  |  |  |  |
| SA_DYS501500 | Deogyusan, Korea | 01-May-14 | *SKK Cho et al. 140501500* | o |  |  |  |  |  |  |  |  |  |  |
| SA_SBK425100 | Jukryeong, Sobaeksan, Korea | 25-Apr-15 | *SKK Cho et al. 150425100* | o | o |  |  |  |  |  |  |  |  |  |
| SA_SBK425110 | Sobaeksan, Korea | 25-Apr-15 | *SKK Cho et al. 150425110* | o | o |  |  |  |  |  |  |  |  |  |
| SA_SBK425112 | Sobaeksan, Korea | 25-Apr-15 | *SKK Cho et al. 150425112* | o | o |  |  |  |  |  |  |  |  |  |
| SA_JRS355-54 | Jirisan, Korea | 12-Apr-13 | *SKK Cho et al. 130412054* | o | o |  |  |  |  |  |  |  |  |  |
| SA_ODS42-112 | Odaesan, Korea | 10-May-14 | *SKK Cho et al. 140510112* | o |  | o |  |  |  |  |  |  |  |  |
| SA_ODS44-19 | Odaesan, Korea | 10-May-14 | *SKK Cho et al. 140510019* | o | o | o |  |  |  |  |  |  |  |  |
| SA_ODS44-21 | Odaesan, Korea | 10-May-14 | *SKK Cho et al. 140510021* | o | o | o |  |  |  |  |  |  |  |  |
| SA_ODS51014 | Odaesan, Korea | 10-May-14 | *SKK Cho et al. 140510014* |  | o |  |  |  |  |  |  |  |  |  |
| SA_ODS51016 | Odaesan, Korea | 10-May-14 | *SKK Cho et al. 140510016* |  | o |  |  |  |  |  |  |  |  |  |
| SA_ODS51017 | Odaesan, Korea | 10-May-14 | *SKK Cho et al. 140510017* |  | o |  |  |  |  |  |  |  |  |  |
| SA_ODS51018 | Odaesan, Korea | 10-May-14 | *SKK Cho et al. 140510018* |  | o |  |  |  |  |  |  |  |  |  |
| SA_ODS51020 | Odaesan, Korea | 10-May-14 | *SKK Cho et al. 140510020* |  | o |  |  |  |  |  |  |  |  |  |
| SA_ODS510111 | Odaesan, Korea | 10-May-14 | *SKK Cho et al. 140510111* |  | o | o |  |  |  |  |  |  |  |  |
| SA_ODS510114 | Odaesan, Korea | 10-May-14 | *SKK Cho et al. 140510114* |  | o | o |  |  |  |  |  |  |  |  |
| SA_ODS510115 | Odaesan, Korea | 10-May-14 | *SKK Cho et al. 140510115* |  | o |  |  |  |  |  |  |  |  |  |
| SA_ODS510118 | Odaesan, Korea | 10-May-14 | *SKK Cho et al. 140510118* |  | o |  |  |  |  |  |  |  |  |  |
| SA_RSS71206 | Botanical Garden Institute FEB RAS, Russia | 12-Jul-14 | *SKK Cho et al. 140712006* | o | o | o |  |  |  |  |  |  |  |  |
| SA_RSS0708 | Primorskiy Krai, Russia | 08-Jul-15 | *SKK Cho et al. 150708* | o | o | o |  |  |  |  |  |  |  |  |
| SA_RSS0706 | Primorskiy Krai, Russia | 06-Jul-15 | *SKK Petrunenko et al. 150706* |  | o |  |  |  |  |  |  |  |  |  |
| SA_RSS0823 | Primorskiy Krai, Russia | 23-Aug-15 | *SKK Pimenova et al. 150823* |  | o |  |  |  |  |  |  |  |  |  |
| SA_RSS803001 | Chekhovo, Sakhalin Oblast, Russia | 03-Aug-15 | *SKK Cho et al. 150803001* | o |  | o |  |  |  |  |  |  |  |  |
| SA_RSS803002 | Chekhovo, Sakhalin Oblast, Russia | 03-Aug-15 | *SKK Cho et al. 150803002* | o |  | o |  |  |  |  |  |  |  |  |
| SA_RSS803003 | Chekhovo, Sakhalin Oblast, Russia | 03-Aug-15 | *SKK Cho et al. 150803003* | o |  | o |  |  |  |  |  |  |  |  |
| PS_1TOHO | Tohoku Univ. Botanical Garden, Sendai, Miyagi, Japan | 20-Apr-12 | None | o |  | o |  |  |  |  |  |  |  |  |
| PS_2-1HIGA | Higashikatsuyama, Sendai, Miyagi, Japan | 26-Apr-12 | None | o |  | o |  |  |  |  |  |  |  |  |
| PS_2-2HIGA | Higashikatsuyama, Sendai, Miyagi, Japan | 27-Apr-12 | None | o |  | o |  |  |  |  |  |  |  |  |
| PS_3MARU | Miyagi, Japan | 29-Apr-12 | None | o |  | o |  |  |  |  |  |  |  |  |
| PS_MYG383-1 | Miyagi, Japan | 23-Apr-13 | *SKK Cho et al. 130423001* | o | o | o |  |  |  |  |  |  |  |  |
| PS_MYG384-8 | Miyagi, Japan | 23-Apr-13 | *SKK Cho et al. 130423008* | o | o | o |  |  |  |  |  |  |  |  |
| PS_MYG42302 | Miyagi, Japan | 23-Apr-13 | *SKK Cho et al. 130423002* |  | o |  |  |  |  |  |  |  |  |  |
| PS_MYG42303 | Miyagi, Japan | 23-Apr-13 | *SKK Cho et al. 130423003* |  | o | o |  |  |  |  |  |  |  |  |
| PS_MYG42304 | Miyagi, Japan | 23-Apr-13 | *SKK Cho et al. 130423004* |  | o | o |  |  |  |  |  |  |  |  |
| PS_MYG42305 | Miyagi, Japan | 23-Apr-13 | *SKK Cho et al. 130423005* |  | o |  |  |  |  |  |  |  |  |  |
| PS_MYG42306 | Miyagi, Japan | 23-Apr-13 | *SKK Cho et al. 130423006* |  | o |  |  |  |  |  |  |  |  |  |
| PS_MYG42307 | Miyagi, Japan | 23-Apr-13 | *SKK Cho et al. 130423007* |  | o |  |  |  |  |  |  |  |  |  |
| PS_MYG42310 | Miyagi, Japan | 23-Apr-13 | *SKK Cho et al. 130423010* |  | o |  |  |  |  |  |  |  |  |  |
| PS_MYG42311 | Miyagi, Japan | 23-Apr-13 | *SKK Cho et al. 130423011* |  | o |  |  |  |  |  |  |  |  |  |
| PS_MYG42314 | Miyagi, Japan | 23-Apr-13 | *SKK Cho et al. 130423014* |  | o |  |  |  |  |  |  |  |  |  |
| PS_MYG42309 | Miyagi, Japan | 23-Apr-13 | *SKK Cho et al. 130423009* |  | o |  |  |  |  |  |  |  |  |  |
| PS_OKA928-200 | Okayama, Japan | 03-Apr-14 | *SKK Cho et al. 140403200* | o |  | o |  |  |  |  |  |  |  |  |
| PS_OKA932-301 | Okayama, Japan | 03-Apr-14 | *SKK Cho et al. 140403301* | o |  | o |  |  |  |  |  |  |  |  |
| PS_OKA403201 | Okayama, Japan | 03-Apr-14 | *SKK Cho et al. 140403201* |  | o | o |  |  |  |  |  |  |  |  |
| PS_OKA403202 | Okayama, Japan | 03-Apr-14 | *SKK Cho et al. 140403202* |  | o | o |  |  |  |  |  |  |  |  |
| PS_OKA403300 | Okayama, Japan | 03-Apr-14 | *SKK Cho et al. 140403300* |  | o | o |  |  |  |  |  |  |  |  |
| PS_OKA403308 | Okayama, Japan | 03-Apr-14 | *SKK Cho et al. 140403308* |  | o |  |  |  |  |  |  |  |  |  |
|  |  |  |  |  |  |  |  |  |  |  |  |  |  |  |
| ***Prunus sargentii* var. *verecunda* (Koidz.) Chin S. Chang, section *Pseudocerasus* Koehne** | | | | | | |  | |  | | |  |  |  |
| VE_YHS42015 | Yeohangsan, Korea |  | *SKK Lim et al. 120420015* |  | o |  |  |  |  |  |  |  |  |  |
| VE_DYS43030 | Deogyusan, Korea |  | *SKK Lim et al. 120430030* |  | o |  |  |  |  |  |  |  |  |  |
| VE_THS50410 | Taehwasan, Korea |  | *SKK Lim et al. 120504010* |  | o |  |  |  |  |  |  |  |  |  |
| VE_SRK51201 | Seoraksan, Korea |  | *SKK Lim et al. 120512001* |  | o |  |  |  |  |  |  |  |  |  |
|  |  |  |  |  |  |  |  |  |  |  |  |  |  |  |
| ***Prunus takesimensis*Nakai, section *Pseudocerasus* Koehne** | |  |  | | | |  |  | |  | | | |  |
| TA_CBU18-001 | Cheonbu, Ulleung Island, Korea | 21-Apr-13 | *SKK Cho et al. 130421001* | o |  | o |  |  |  |  |  |  |  |  |
| TA_CBU18-19 | Cheonbu, Ulleung Island, Korea | 21-Apr-13 | *SKK Cho et al. 130421019* | o |  | o |  |  |  |  |  |  |  |  |
| TA_CBU530-129 | Cheonbu, Ulleung Island, Korea | 10-Aug-13 | None | o |  | o |  |  |  |  |  |  |  |  |
| TA_CBU42545 | Cheonbu, Ulleung Island, Korea | 25-Apr-16 | *SKK Cho et al. 160425045* |  | o |  |  |  |  |  |  |  |  |  |
| TA_CBU42546 | Cheonbu, Ulleung Island, Korea | 25-Apr-16 | *SKK Cho et al. 160425046* |  | o | o |  |  |  |  |  |  |  |  |
| TA_CBU42547 | Cheonbu, Ulleung Island, Korea | 25-Apr-16 | *SKK Cho et al. 160425047* |  | o |  |  |  |  |  |  |  |  |  |
| TA_CBU42549 | Cheonbu, Ulleung Island, Korea | 25-Apr-16 | *SKK Cho et al. 160425049* |  | o |  |  |  |  |  |  |  |  |  |
| TA_CBU42550 | Cheonbu, Ulleung Island, Korea | 25-Apr-16 | *SKK Cho et al. 160425050* |  | o |  |  |  |  |  |  |  |  |  |
| TA_CBU42519 | Cheonbu, Ulleung Island, Korea | 25-Apr-16 | *SKK Cho et al. 160425119* |  | o |  |  |  |  |  |  |  |  |  |
| TA_CBU42520 | Cheonbu, Ulleung Island, Korea | 25-Apr-16 | *SKK Cho et al. 160425120* |  | o |  |  |  |  |  |  |  |  |  |
| TA_CBU42521 | Cheonbu, Ulleung Island, Korea | 25-Apr-16 | *SKK Cho et al. 160425121* |  | o |  |  |  |  |  |  |  |  |  |
| TA_CBU42522 | Cheonbu, Ulleung Island, Korea | 25-Apr-16 | *SKK Cho et al. 160425122* |  | o |  |  |  |  |  |  |  |  |  |
| TA_CBU42523 | Cheonbu, Ulleung Island, Korea | 25-Apr-16 | *SKK Cho et al. 160425123* |  | o | o |  |  |  |  |  |  |  |  |
| TA_CHU42324 | Chusan, Ulleung Island, Korea | 23-Apr-16 | *SKK Cho et al. 160423024* |  | o |  |  |  |  |  |  |  |  |  |
| TA_CHU42325 | Chusan, Ulleung Island, Korea | 23-Apr-16 | *SKK Cho et al. 160423025* |  | o |  |  |  |  |  |  |  |  |  |
| TA_CHU42326 | Chusan, Ulleung Island, Korea | 23-Apr-16 | *SKK Cho et al. 160423026* |  | o | o |  |  |  |  |  |  |  |  |
| TA_CHU42327 | Chusan, Ulleung Island, Korea | 23-Apr-16 | *SKK Cho et al. 160423027* |  | o |  |  |  |  |  |  |  |  |  |
| TA_CHU42328 | Chusan, Ulleung Island, Korea | 23-Apr-16 | *SKK Cho et al. 160423028* |  | o |  |  |  |  |  |  |  |  |  |
| TA_CHU42329 | Chusan, Ulleung Island, Korea | 23-Apr-16 | *SKK Cho et al. 160423029* |  | o |  |  |  |  |  |  |  |  |  |
| TA_CHU42330 | Chusan, Ulleung Island, Korea | 23-Apr-16 | *SKK Cho et al. 160423030* |  | o | o |  |  |  |  |  |  |  |  |
| TA_CHU42331 | Chusan, Ulleung Island, Korea | 23-Apr-16 | *SKK Cho et al. 160423031* |  | o |  |  |  |  |  |  |  |  |  |
| TA_CHU42332 | Chusan, Ulleung Island, Korea | 23-Apr-16 | *SKK Cho et al. 160423032* |  | o |  |  |  |  |  |  |  |  |  |
| TA_CHU42333 | Chusan, Ulleung Island, Korea | 23-Apr-16 | *SKK Cho et al. 160423033* |  | o | o |  |  |  |  |  |  |  |  |
| TA_CHU42335 | Chusan, Ulleung Island, Korea | 23-Apr-16 | *SKK Cho et al. 160423035* |  | o | o |  |  |  |  |  |  |  |  |
| TA_CHU42336 | Chusan, Ulleung Island, Korea | 23-Apr-16 | *SKK Cho et al. 160423036* |  | o |  |  |  |  |  |  |  |  |  |
| TA_CHU42337 | Chusan, Ulleung Island, Korea | 23-Apr-16 | *SKK Cho et al. 160423037* |  | o |  |  |  |  |  |  |  |  |  |
| TA_CHU42338 | Chusan, Ulleung Island, Korea | 23-Apr-16 | *SKK Cho et al. 160423038* |  | o |  |  |  |  |  |  |  |  |  |
| TA_DDJ515-125 | Dokdo Jeonmangdae, Ulleung Island, Korea | 09-Aug-13 | None | o |  | o |  |  |  |  |  |  |  |  |
| TA_DDJ515-126 | Dokdo Jeonmangdae, Ulleung Island, Korea | 09-Aug-13 | None | o |  | o |  |  |  |  |  |  |  |  |
| TA_DDJ21-63 | Dokdo Jeonmangdae, Ulleung Island, Korea | 22-Apr-13 | *SKK Cho et al. 130422063* | o |  | o |  |  |  |  |  |  |  |  |
| TA_DDJ21-68 | Dokdo Jeonmangdae, Ulleung Island, Korea | 22-Apr-13 | *SKK Cho et al. 130422068* | o |  | o |  |  |  |  |  |  |  |  |
| TA_DDJ42308 | Dokdo Jeonmangdae, Ulleung Island, Korea | 23-Apr-16 | *SKK Cho et al. 160423008* |  | o | o |  |  |  |  |  |  |  |  |
| TA_DDJ42309 | Dokdo Jeonmangdae, Ulleung Island, Korea | 23-Apr-16 | *SKK Cho et al. 160423009* |  | o |  |  |  |  |  |  |  |  |  |
| TA_DDJ42310 | Dokdo Jeonmangdae, Ulleung Island, Korea | 23-Apr-16 | *SKK Cho et al. 160423010* |  | o |  |  |  |  |  |  |  |  |  |
| TA_DDJ42312 | Dokdo Jeonmangdae, Ulleung Island, Korea | 23-Apr-16 | *SKK Cho et al. 160423012* |  | o |  |  |  |  |  |  |  |  |  |
| TA_DDJ42313 | Dokdo Jeonmangdae, Ulleung Island, Korea | 23-Apr-16 | *SKK Cho et al. 160423013* |  | o |  |  |  |  |  |  |  |  |  |
| TA_DDJ42314 | Dokdo Jeonmangdae, Ulleung Island, Korea | 23-Apr-16 | *SKK Cho et al. 160423014* |  | o |  |  |  |  |  |  |  |  |  |
| TA_DDJ42316 | Dokdo Jeonmangdae, Ulleung Island, Korea | 23-Apr-16 | *SKK Cho et al. 160423016* |  | o |  |  |  |  |  |  |  |  |  |
| TA_DDJ42317 | Dokdo Jeonmangdae, Ulleung Island, Korea | 23-Apr-16 | *SKK Cho et al. 160423017* |  | o |  |  |  |  |  |  |  |  |  |
| TA_DDJ42318 | Dokdo Jeonmangdae, Ulleung Island, Korea | 23-Apr-16 | *SKK Cho et al. 160423018* |  | o |  |  |  |  |  |  |  |  |  |
| TA_DDJ42320 | Dokdo Jeonmangdae, Ulleung Island, Korea | 23-Apr-16 | *SKK Cho et al. 160423020* |  | o |  |  |  |  |  |  |  |  |  |
| TA_DDJ42321 | Dokdo Jeonmangdae, Ulleung Island, Korea | 23-Apr-16 | *SKK Cho et al. 160423021* |  | o |  |  |  |  |  |  |  |  |  |
| TA_DDJ42322 | Dokdo Jeonmangdae, Ulleung Island, Korea | 23-Apr-16 | *SKK Cho et al. 160423022* |  | o |  |  |  |  |  |  |  |  |  |
| TA_HGM42551 | Hyangmok Jeonmangdae, Ulleung Island, Korea | 25-Apr-16 | *SKK Cho et al. 160425051* |  | o |  |  |  |  |  |  |  |  |  |
| TA_HGM42552 | Hyangmok Jeonmangdae, Ulleung Island, Korea | 25-Apr-16 | *SKK Cho et al. 160425052* |  | o |  |  |  |  |  |  |  |  |  |
| TA_HGM42553 | Hyangmok Jeonmangdae, Ulleung Island, Korea | 25-Apr-16 | *SKK Cho et al. 160425053* |  | o |  |  |  |  |  |  |  |  |  |
| TA_HGM42554 | Hyangmok Jeonmangdae, Ulleung Island, Korea | 25-Apr-16 | *SKK Cho et al. 160425054* |  | o |  |  |  |  |  |  |  |  |  |
| TA_HGM42555 | Hyangmok Jeonmangdae, Ulleung Island, Korea | 25-Apr-16 | *SKK Cho et al. 160425055* |  | o |  |  |  |  |  |  |  |  |  |
| TA_HGM42556 | Hyangmok Jeonmangdae, Ulleung Island, Korea | 25-Apr-16 | *SKK Cho et al. 160425056* |  | o | o |  |  |  |  |  |  |  |  |
| TA_HGM42557 | Hyangmok Jeonmangdae, Ulleung Island, Korea | 25-Apr-16 | *SKK Cho et al. 160425057* |  | o |  |  |  |  |  |  |  |  |  |
| TA_HGM42558 | Hyangmok Jeonmangdae, Ulleung Island, Korea | 25-Apr-16 | *SKK Cho et al. 160425058* |  | o | o |  |  |  |  |  |  |  |  |
| TA_HGM42559 | Hyangmok Jeonmangdae, Ulleung Island, Korea | 25-Apr-16 | *SKK Cho et al. 160425059* |  | o |  |  |  |  |  |  |  |  |  |
| TA_HGM42524 | Hyangmok Jeonmangdae, Ulleung Island, Korea | 25-Apr-16 | *SKK Cho et al. 160425124* |  | o |  |  |  |  |  |  |  |  |  |
| TA_HGM42525 | Hyangmok Jeonmangdae, Ulleung Island, Korea | 25-Apr-16 | *SKK Cho et al. 160425125* |  | o | o |  |  |  |  |  |  |  |  |
| TA_HGM42526 | Hyangmok Jeonmangdae, Ulleung Island, Korea | 25-Apr-16 | *SKK Cho et al. 160425126* |  | o | o |  |  |  |  |  |  |  |  |
| TA_HGM42527 | Hyangmok Jeonmangdae, Ulleung Island, Korea | 25-Apr-16 | *SKK Cho et al. 160425127* |  | o |  |  |  |  |  |  |  |  |  |
| TA_HGM42528 | Hyangmok Jeonmangdae, Ulleung Island, Korea | 25-Apr-16 | *SKK Cho et al. 160425128* |  | o | o |  |  |  |  |  |  |  |  |
| TA_HPR534-232 | Hyunpo-ri, Ulleung Island, Korea | 10-Aug-13 | None | o | o | o |  |  |  |  |  |  |  |  |
| TA_HPR534-241 | Hyunpo-ri, Ulleung Island, Korea | 10-Aug-13 | None | o |  | o |  |  |  |  |  |  |  |  |
| TA_HPR534-245 | Hyunpo-ri, Ulleung Island, Korea | 10-Aug-13 | None | o |  | o |  |  |  |  |  |  |  |  |
| TA_HPR42229 | Hyunpo-ri, Ulleung Island, Korea | 22-Apr-16 | *SKK Cho et al. 160422029* |  | o | o |  |  |  |  |  |  |  |  |
| TA_HPR42230 | Hyunpo-ri, Ulleung Island, Korea | 22-Apr-16 | *SKK Cho et al. 160422030* |  | o |  |  |  |  |  |  |  |  |  |
| TA_HPR42231 | Hyunpo-ri, Ulleung Island, Korea | 22-Apr-16 | *SKK Cho et al. 160422031* |  | o |  |  |  |  |  |  |  |  |  |
| TA_HPR42232 | Hyunpo-ri, Ulleung Island, Korea | 22-Apr-16 | *SKK Cho et al. 160422032* |  | o |  |  |  |  |  |  |  |  |  |
| TA_HPR42233 | Hyunpo-ri, Ulleung Island, Korea | 22-Apr-16 | *SKK Cho et al. 160422033* |  | o |  |  |  |  |  |  |  |  |  |
| TA_HPR42234 | Hyunpo-ri, Ulleung Island, Korea | 22-Apr-16 | *SKK Cho et al. 160422034* |  | o |  |  |  |  |  |  |  |  |  |
| TA_HPR42235 | Hyunpo-ri, Ulleung Island, Korea | 22-Apr-16 | *SKK Cho et al. 160422035* |  | o |  |  |  |  |  |  |  |  |  |
| TA_HPR42236 | Hyunpo-ri, Ulleung Island, Korea | 22-Apr-16 | *SKK Cho et al. 160422036* |  | o | o |  |  |  |  |  |  |  |  |
| TA_HPR42237 | Hyunpo-ri, Ulleung Island, Korea | 22-Apr-16 | *SKK Cho et al. 160422037* |  | o |  |  |  |  |  |  |  |  |  |
| TA_HPR42238 | Hyunpo-ri, Ulleung Island, Korea | 22-Apr-16 | *SKK Cho et al. 160422038* |  | o |  |  |  |  |  |  |  |  |  |
| TA_HPR42239 | Hyunpo-ri, Ulleung Island, Korea | 22-Apr-16 | *SKK Cho et al. 160422039* |  | o |  |  |  |  |  |  |  |  |  |
| TA_JRG42211 | Joongryong, Ulleung Island, Korea | 22-Apr-16 | *SKK Cho et al. 160422011* |  | o | o |  |  |  |  |  |  |  |  |
| TA_JRG42212 | Joongryong, Ulleung Island, Korea | 22-Apr-16 | *SKK Cho et al. 160422012* |  | o |  |  |  |  |  |  |  |  |  |
| TA_JRG42213 | Joongryong, Ulleung Island, Korea | 22-Apr-16 | *SKK Cho et al. 160422013* |  | o |  |  |  |  |  |  |  |  |  |
| TA_JRG42214 | Joongryong, Ulleung Island, Korea | 22-Apr-16 | *SKK Cho et al. 160422014* |  | o |  |  |  |  |  |  |  |  |  |
| TA_JRG42215 | Joongryong, Ulleung Island, Korea | 22-Apr-16 | *SKK Cho et al. 160422015* |  | o | o |  |  |  |  |  |  |  |  |
| TA_JRG42216 | Joongryong, Ulleung Island, Korea | 22-Apr-16 | *SKK Cho et al. 160422016* |  | o | o |  |  |  |  |  |  |  |  |
| TA_JRG42217 | Joongryong, Ulleung Island, Korea | 22-Apr-16 | *SKK Cho et al. 160422017* |  | o |  |  |  |  |  |  |  |  |  |
| TA_JRG42221 | Joongryong, Ulleung Island, Korea | 22-Apr-16 | *SKK Cho et al. 160422021* |  | o |  |  |  |  |  |  |  |  |  |
| TA_JRG42222 | Joongryong, Ulleung Island, Korea | 22-Apr-16 | *SKK Cho et al. 160422022* |  | o |  |  |  |  |  |  |  |  |  |
| TA_JRG42223 | Joongryong, Ulleung Island, Korea | 22-Apr-16 | *SKK Cho et al. 160422023* |  | o |  |  |  |  |  |  |  |  |  |
| TA_JRG42224 | Joongryong, Ulleung Island, Korea | 22-Apr-16 | *SKK Cho et al. 160422024* |  | o |  |  |  |  |  |  |  |  |  |
| TA_JRG42225 | Joongryong, Ulleung Island, Korea | 22-Apr-16 | *SKK Cho et al. 160422025* |  | o |  |  |  |  |  |  |  |  |  |
| TA_JRG42226 | Joongryong, Ulleung Island, Korea | 22-Apr-16 | *SKK Cho et al. 160422026* |  | o | o |  |  |  |  |  |  |  |  |
| TA_JRG42227 | Joongryong, Ulleung Island, Korea | 22-Apr-16 | *SKK Cho et al. 160422027* |  | o | o |  |  |  |  |  |  |  |  |
| TA_MAL42410 | Maljandeung, Ulleung Island, Korea | 24-Apr-16 | *SKK Cho et al. 160424010* |  | o | o |  |  |  |  |  |  |  |  |
| TA_MAL42411 | Maljandeung, Ulleung Island, Korea | 24-Apr-16 | *SKK Cho et al. 160424011* |  | o | o |  |  |  |  |  |  |  |  |
| TA_MAL42412 | Maljandeung, Ulleung Island, Korea | 24-Apr-16 | *SKK Cho et al. 160424012* |  | o |  |  |  |  |  |  |  |  |  |
| TA_MAL42413 | Maljandeung, Ulleung Island, Korea | 24-Apr-16 | *SKK Cho et al. 160424013* |  | o | o |  |  |  |  |  |  |  |  |
| TA_MAL42414 | Maljandeung, Ulleung Island, Korea | 24-Apr-16 | *SKK Cho et al. 160424014* |  | o | o |  |  |  |  |  |  |  |  |
| TA_MAL42416 | Maljandeung, Ulleung Island, Korea | 24-Apr-16 | *SKK Cho et al. 160424016* |  | o |  |  |  |  |  |  |  |  |  |
| TA_MAL42417 | Maljandeung, Ulleung Island, Korea | 24-Apr-16 | *SKK Cho et al. 160424017* |  | o |  |  |  |  |  |  |  |  |  |
| TA_MAL42418 | Maljandeung, Ulleung Island, Korea | 24-Apr-16 | *SKK Cho et al. 160424018* |  | o |  |  |  |  |  |  |  |  |  |
| TA_MAL42419 | Maljandeung, Ulleung Island, Korea | 24-Apr-16 | *SKK Cho et al. 160424019* |  | o |  |  |  |  |  |  |  |  |  |
| TA_MAL42420 | Maljandeung, Ulleung Island, Korea | 24-Apr-16 | *SKK Cho et al. 160424020* |  | o | o |  |  |  |  |  |  |  |  |
| TA_NAM28-158 | Namseo-ri, Ulleung Island, Korea | 23-Apr-13 | *SKK Cho et al. 130423158* | o |  | o |  |  |  |  |  |  |  |  |
| TA_NAM33-209 | Namseo-ri, Ulleung Island, Korea | 24-Apr-13 | *SKK Cho et al. 130424209* | o |  | o |  |  |  |  |  |  |  |  |
| TA_NAM555-110 | Namseo-ri, Ulleung Island, Korea | 11-Aug-13 | None | o |  | o |  |  |  |  |  |  |  |  |
| TA_NAM555-111 | Namseo-ri, Ulleung Island, Korea | 11-Aug-13 | None | o |  | o |  |  |  |  |  |  |  |  |
| TA_NAM42560 | Namseo-ri, Ulleung Island, Korea | 25-Apr-16 | *SKK Cho et al. 160425060* |  | o |  |  |  |  |  |  |  |  |  |
| TA_NAM42561 | Namseo-ri, Ulleung Island, Korea | 25-Apr-16 | *SKK Cho et al. 160425061* |  | o |  |  |  |  |  |  |  |  |  |
| TA_NAM42563 | Namseo-ri, Ulleung Island, Korea | 25-Apr-16 | *SKK Cho et al. 160425063* |  | o |  |  |  |  |  |  |  |  |  |
| TA_NAM42564 | Namseo-ri, Ulleung Island, Korea | 25-Apr-16 | *SKK Cho et al. 160425064* |  | o |  |  |  |  |  |  |  |  |  |
| TA_NAM42565 | Namseo-ri, Ulleung Island, Korea | 25-Apr-16 | *SKK Cho et al. 160425065* |  | o |  |  |  |  |  |  |  |  |  |
| TA_NAM42566 | Namseo-ri, Ulleung Island, Korea | 25-Apr-16 | *SKK Cho et al. 160425066* |  | o |  |  |  |  |  |  |  |  |  |
| TA_NAM42567 | Namseo-ri, Ulleung Island, Korea | 25-Apr-16 | *SKK Cho et al. 160425067* |  | o |  |  |  |  |  |  |  |  |  |
| TA_NAM42568 | Namseo-ri, Ulleung Island, Korea | 25-Apr-16 | *SKK Cho et al. 160425068* |  | o |  |  |  |  |  |  |  |  |  |
| TA_NAM42529 | Namseo-ri, Ulleung Island, Korea | 25-Apr-16 | *SKK Cho et al. 160425129* |  | o | o |  |  |  |  |  |  |  |  |
| TA_NAR521-116 | Nari, Ulleung Island, Korea | 10-Aug-13 | None | o |  | o |  |  |  |  |  |  |  |  |
| TA_NAR524-117 | Nari, Ulleung Island, Korea | 10-Aug-13 | None | o | o | o |  |  |  |  |  |  |  |  |
| TA_NAR524-139 | Nari, Ulleung Island, Korea | 10-Aug-13 | None | o |  | o |  |  |  |  |  |  |  |  |
| TA_NAR25-116 | Nari, Ulleung Island, Korea | 23-Apr-13 | *SKK Cho et al. 130423116* | o |  | o |  |  |  |  |  |  |  |  |
| TA_NAR26-129 | Nari, Ulleung Island, Korea | 23-Apr-13 | *SKK Cho et al. 130423129* | o |  | o |  |  |  |  |  |  |  |  |
| TA_NAR27-135 | Nari, Ulleung Island, Korea | 23-Apr-13 | *SKK Cho et al. 130423135* | o |  | o |  |  |  |  |  |  |  |  |
| TA_NAR42520 | Nari, Ulleung Island, Korea | 25-Apr-16 | *SKK Cho et al. 160425020* |  | o |  |  |  |  |  |  |  |  |  |
| TA_NAR42521 | Nari, Ulleung Island, Korea | 25-Apr-16 | *SKK Cho et al. 160425021* |  | o |  |  |  |  |  |  |  |  |  |
| TA_NAR42523 | Nari, Ulleung Island, Korea | 25-Apr-16 | *SKK Cho et al. 160425023* |  | o |  |  |  |  |  |  |  |  |  |
| TA_NAR42524 | Nari, Ulleung Island, Korea | 25-Apr-16 | *SKK Cho et al. 160425024* |  | o |  |  |  |  |  |  |  |  |  |
| TA_NAR42526 | Nari, Ulleung Island, Korea | 25-Apr-16 | *SKK Cho et al. 160425026* |  | o |  |  |  |  |  |  |  |  |  |
| TA_NAR42527 | Nari, Ulleung Island, Korea | 25-Apr-16 | *SKK Cho et al. 160425027* |  | o |  |  |  |  |  |  |  |  |  |
| TA_NAR42534 | Nari, Ulleung Island, Korea | 25-Apr-16 | *SKK Cho et al. 160425034* |  | o |  |  |  |  |  |  |  |  |  |
| TA_NAR42535 | Nari, Ulleung Island, Korea | 25-Apr-16 | *SKK Cho et al. 160425035* |  | o |  |  |  |  |  |  |  |  |  |
| TA_NAR42536 | Nari, Ulleung Island, Korea | 25-Apr-16 | *SKK Cho et al. 160425036* |  | o |  |  |  |  |  |  |  |  |  |
| TA_NAR425103 | Nari, Ulleung Island, Korea | 25-Apr-16 | *SKK Cho et al. 160425103* |  | o |  |  |  |  |  |  |  |  |  |
| TA_NRH42504 | Nari, Ulleung Island, Korea | 25-Apr-16 | *SKK Cho et al. 160425004* |  | o |  |  |  |  |  |  |  |  |  |
| TA_NRH42506 | Nari, Ulleung Island, Korea | 25-Apr-16 | *SKK Cho et al. 160425006* |  | o |  |  |  |  |  |  |  |  |  |
| TA_NRH42507 | Nari, Ulleung Island, Korea | 25-Apr-16 | *SKK Cho et al. 160425007* |  | o |  |  |  |  |  |  |  |  |  |
| TA_NRH42508 | Nari, Ulleung Island, Korea | 25-Apr-16 | *SKK Cho et al. 160425008* |  | o |  |  |  |  |  |  |  |  |  |
| TA_NRH42531 | Nari, Ulleung Island, Korea | 25-Apr-16 | *SKK Cho et al. 160425031* |  | o |  |  |  |  |  |  |  |  |  |
| TA_NRH42532 | Nari, Ulleung Island, Korea | 25-Apr-16 | *SKK Cho et al. 160425032* |  | o |  |  |  |  |  |  |  |  |  |
| TA_NRH42539 | Nari, Ulleung Island, Korea | 25-Apr-16 | *SKK Cho et al. 160425039* |  | o |  |  |  |  |  |  |  |  |  |
| TA_NRH42543 | Nari, Ulleung Island, Korea | 25-Apr-16 | *SKK Cho et al. 160425043* |  | o |  |  |  |  |  |  |  |  |  |
| TA_NRH425102 | Nari, Ulleung Island, Korea | 25-Apr-16 | *SKK Cho et al. 160425102* |  | o |  |  |  |  |  |  |  |  |  |
| TA_NRH425104 | Nari, Ulleung Island, Korea | 25-Apr-16 | *SKK Cho et al. 160425104* |  | o |  |  |  |  |  |  |  |  |  |
| TA_NSJ20-37 | Naesujeon, Ulleung Island, Korea | 22-Apr-13 | *SKK Cho et al. 130422037* | o |  | o |  |  |  |  |  |  |  |  |
| TA_NSJ42001 | Naesujeon, Ulleung Island, Korea | 20-Apr-16 | *SKK Cho et al. 160420001* |  |  | o |  |  |  |  |  |  |  |  |
| TA_NSJ42002 | Naesujeon, Ulleung Island, Korea | 20-Apr-16 | *SKK Cho et al. 160420002* |  | o | o |  |  |  |  |  |  |  |  |
| TA_NSJ42013 | Naesujeon, Ulleung Island, Korea | 20-Apr-16 | *SKK Cho et al. 160420013* |  | o |  |  |  |  |  |  |  |  |  |
| TA_NSJ42003 | Naesujeon, Ulleung Island, Korea | 20-Apr-16 | *SKK Cho et al. 160420003* |  | o |  |  |  |  |  |  |  |  |  |
| TA_NSJ42006 | Naesujeon, Ulleung Island, Korea | 20-Apr-16 | *SKK Cho et al. 160420006* |  | o |  |  |  |  |  |  |  |  |  |
| TA_NSJ42010 | Naesujeon, Ulleung Island, Korea | 20-Apr-16 | *SKK Cho et al. 160420010* |  | o |  |  |  |  |  |  |  |  |  |
| TA_SAD32-197 | Sa-dong, Ulleung Island, Korea | 23-Apr-13 | *SKK Cho et al. 130423197* | o |  | o |  |  |  |  |  |  |  |  |
| TA_SAD42201 | Sa-dong, Ulleung Island, Korea | 22-Apr-16 | *SKK Cho et al. 160422001* |  | o | o |  |  |  |  |  |  |  |  |
| TA_SAD42202 | Sa-dong, Ulleung Island, Korea | 22-Apr-16 | *SKK Cho et al. 160422002* |  | o | o |  |  |  |  |  |  |  |  |
| TA_SAD42203 | Sa-dong, Ulleung Island, Korea | 22-Apr-16 | *SKK Cho et al. 160422003* |  | o |  |  |  |  |  |  |  |  |  |
| TA_SAD42204 | Sa-dong, Ulleung Island, Korea | 22-Apr-16 | *SKK Cho et al. 160422004* |  | o |  |  |  |  |  |  |  |  |  |
| TA_SAD42206 | Sa-dong, Ulleung Island, Korea | 22-Apr-16 | *SKK Cho et al. 160422006* |  | o |  |  |  |  |  |  |  |  |  |
| TA_SAD42207 | Sa-dong, Ulleung Island, Korea | 22-Apr-16 | *SKK Cho et al. 160422007* |  | o |  |  |  |  |  |  |  |  |  |
| TA_SAD42208 | Sa-dong, Ulleung Island, Korea | 22-Apr-16 | *SKK Cho et al. 160422008* |  | o | o |  |  |  |  |  |  |  |  |
| TA_SAD42209 | Sa-dong, Ulleung Island, Korea | 22-Apr-16 | *SKK Cho et al. 160422009* |  | o | o |  |  |  |  |  |  |  |  |
| TA_SAD42210 | Sa-dong, Ulleung Island, Korea | 22-Apr-16 | *SKK Cho et al. 160422010* |  | o |  |  |  |  |  |  |  |  |  |
| TA_SIB2115-1 | Seonginbong, Ulleung Island, Korea | 24-Apr-14 | None | o |  | o |  |  |  |  |  |  |  |  |
| TA_SIB2115-6 | Seonginbong, Ulleung Island, Korea | 24-Apr-14 | None | o |  | o |  |  |  |  |  |  |  |  |
| TA_SIB969-002 | Seonginbong, Ulleung Island, Korea | 24-Apr-14 | None | o | o | o |  |  |  |  |  |  |  |  |
| TA_SIB969-004 | Seonginbong, Ulleung Island, Korea | 24-Apr-14 | None | o | o | o |  |  |  |  |  |  |  |  |
| TA_SIB969-005 | Seonginbong, Ulleung Island, Korea | 24-Apr-14 | None |  | o |  |  |  |  |  |  |  |  |  |
| TA_SIB969-006 | Seonginbong, Ulleung Island, Korea | 24-Apr-14 | None | o | o | o |  |  |  |  |  |  |  |  |
| TA_SIB42453 | Seonginbong, Ulleung Island, Korea | 24-Apr-16 | *SKK Cho et al. 160424053* |  | o |  |  |  |  |  |  |  |  |  |
| TA_SIB42454 | Seonginbong, Ulleung Island, Korea | 24-Apr-16 | *SKK Cho et al. 160424054* |  | o | o |  |  |  |  |  |  |  |  |
| TA_SIB42455 | Seonginbong, Ulleung Island, Korea | 24-Apr-16 | *SKK Cho et al. 160424055* |  | o | o |  |  |  |  |  |  |  |  |
| TA_SIB42456 | Seonginbong, Ulleung Island, Korea | 24-Apr-16 | *SKK Cho et al. 160424056* |  | o |  |  |  |  |  |  |  |  |  |
| TA_SIB42457 | Seonginbong, Ulleung Island, Korea | 24-Apr-16 | *SKK Cho et al. 160424057* |  | o |  |  |  |  |  |  |  |  |  |
| TA_SIB42458 | Seonginbong, Ulleung Island, Korea | 24-Apr-16 | *SKK Cho et al. 160424058* |  | o | o |  |  |  |  |  |  |  |  |
| TA_SIB42459 | Seonginbong, Ulleung Island, Korea | 24-Apr-16 | *SKK Cho et al. 160424059* |  | o |  |  |  |  |  |  |  |  |  |
| TA_SIB42460 | Seonginbong, Ulleung Island, Korea | 24-Apr-16 | *SKK Cho et al. 160424060* |  | o |  |  |  |  |  |  |  |  |  |
| TA_SIB42461 | Seonginbong, Ulleung Island, Korea | 24-Apr-16 | *SKK Cho et al. 160424061* |  | o |  |  |  |  |  |  |  |  |  |
| TA_SIB42462 | Seonginbong, Ulleung Island, Korea | 24-Apr-16 | *SKK Cho et al. 160424062* |  | o |  |  |  |  |  |  |  |  |  |
| TA_SIB42463 | Seonginbong, Ulleung Island, Korea | 24-Apr-16 | *SKK Cho et al. 160424063* |  | o |  |  |  |  |  |  |  |  |  |
| TA_SIB42464 | Seonginbong, Ulleung Island, Korea | 24-Apr-16 | *SKK Cho et al. 160424064* |  | o |  |  |  |  |  |  |  |  |  |
| TA_SIB42465 | Seonginbong, Ulleung Island, Korea | 24-Apr-16 | *SKK Cho et al. 160424065* |  | o |  |  |  |  |  |  |  |  |  |
| TA_SIB42466 | Seonginbong, Ulleung Island, Korea | 24-Apr-16 | *SKK Cho et al. 160424066* |  | o |  |  |  |  |  |  |  |  |  |
| TA_SIB42467 | Seonginbong, Ulleung Island, Korea | 24-Apr-16 | *SKK Cho et al. 160424067* |  | o |  |  |  |  |  |  |  |  |  |
| TA_THR24-101 | Taeha-ri, Ulleung Island, Korea | 23-Apr-13 | *SKK Cho et al. 130423101* | o |  | o |  |  |  |  |  |  |  |  |
| TA_THR42109 | Taeha-ri, Ulleung Island, Korea | 21-Apr-16 | *SKK Cho et al. 160421009* |  | o | o |  |  |  |  |  |  |  |  |
| TA_THR42110 | Taeha-ri, Ulleung Island, Korea | 21-Apr-16 | *SKK Cho et al. 160421010* |  | o | o |  |  |  |  |  |  |  |  |
| TA_THR42111 | Taeha-ri, Ulleung Island, Korea | 21-Apr-16 | *SKK Cho et al. 160421011* |  | o | o |  |  |  |  |  |  |  |  |
| TA_THR42112 | Taeha-ri, Ulleung Island, Korea | 21-Apr-16 | *SKK Cho et al. 160421012* |  | o | o |  |  |  |  |  |  |  |  |
| TA_THR42113 | Taeha-ri, Ulleung Island, Korea | 21-Apr-16 | *SKK Cho et al. 160421013* |  | o |  |  |  |  |  |  |  |  |  |
| TA_THR42114 | Taeha-ri, Ulleung Island, Korea | 21-Apr-16 | *SKK Cho et al. 160421014* |  | o |  |  |  |  |  |  |  |  |  |
| TA_THR42115 | Taeha-ri, Ulleung Island, Korea | 21-Apr-16 | *SKK Cho et al. 160421015* |  | o |  |  |  |  |  |  |  |  |  |
| TA_THR42116 | Taeha-ri, Ulleung Island, Korea | 21-Apr-16 | *SKK Cho et al. 160421016* |  | o |  |  |  |  |  |  |  |  |  |
| TA_THR42117 | Taeha-ri, Ulleung Island, Korea | 21-Apr-16 | *SKK Cho et al. 160421017* |  | o |  |  |  |  |  |  |  |  |  |
| TA_THR42118 | Taeha-ri, Ulleung Island, Korea | 21-Apr-16 | *SKK Cho et al. 160421018* |  | o |  |  |  |  |  |  |  |  |  |
| TA_DDR514-145 | Do-dong, Ulleung Island, Korea | 09-Aug-13 | None | o |  |  |  |  |  |  |  |  |  |  |
| TA_DDR42016 | Do-dong, Ulleung Island, Korea | 20-Apr-16 | *SKK Cho et al. 160420016* |  | o |  |  |  |  |  |  |  |  |  |
| TA_DDR540-292 | Do-dong, Ulleung Island, Korea | 11-Aug-13 | None | o | o |  |  |  |  |  |  |  |  |  |
| TA_DDR540-294 | Do-dong, Ulleung Island, Korea | 11-Aug-13 | None | o |  |  |  |  |  |  |  |  |  |  |
| TA_BRF537-244 | Bongrae Falls, Ulleung Island, Korea | 11-Aug-13 | None | o | o |  |  |  |  |  |  |  |  |  |
| TA_BRF23-80 | Bongrae Falls, Ulleung Island, Korea | 22-Apr-13 | *SKK Cho et al. 130422080* | o |  |  |  |  |  |  |  |  |  |  |
| TA_NYR549-169 | Namyang-ri, Ulleung Island, Korea | 11-Aug-13 | None | o |  |  |  |  |  |  |  |  |  |  |

**Table S2.** Summary of bootstrap supports in the phylogenetic analyses based on variable parameters used in STACKS analyses. Population codes of *P. sargentii* are labelled as KP, RSS, MYG, OKA and JJ for the populations of Korean Peninsula, Russia, Miyagi, Okayama and Jeju Island, respectively. The bootstrap support values above 90% are shown in bold.

| r 0.75 applied for all p values |  |  |  |  |  |  |
| --- | --- | --- | --- | --- | --- | --- |
| p value | 2 | 8 | 16 | 24 | 32 | 40 |
| Number of loci | 9027 | 3889 | 1980 | 1148 | 806 | 572 |
| Number of SNP | 13063 | 5899 | 2898 | 1597 | 1098 | 769 |
| Bootstrap support values for major clades (%) | |  |  |  |  |  |
| *P. takesimensis* | **100** | **100** | **100** | **100** | **99** | **100** |
| CLADE A including *P. takesimensis* and the KP, RSS and MYG populations of *P. sargentii* | 76 | **97** | **90** | **91** | 66 | **98** |
| *P. speciosa* | **97** | **98** | **100** | **100** | **99** | **99** |
| *P. sargentii* |  |  |  |  |  |  |
| population KP+RSS | 84 | **90** | 55 | **92** | 67 | **93** |
| population MYG | **99** | **100** | 50 | **99** | **96** | 85 |
| population OKA | **91** | **93** | **92** | **95** | 85 | 80 |
| population JJ | **98** | **94** | 83 | 79 | 67 | 56 |
| Average bootstrap value (%) | **92** | **96** | 81 | **94** | 83 | 88 |
